# Rolapitant treats lung cancer by targeting deubiquitinase OTUD3

**DOI:** 10.1186/s12964-024-01519-8

**Published:** 2024-03-27

**Authors:** Tongde Du, Quan Gu, Yonghui Zhang, Yujie Gan, Rongrui Liang, Wenzhu Yang, Ya Lu, Chenxin Xu, Jianzhong Wu, Rong Ma, Haixia Cao, Jingwei Jiang, Juan Wang, Jifeng Feng

**Affiliations:** 1 Jiangsu Cancer Hospital, Jiangsu Institute of Cancer Research, The Affiliated Cancer Hospital of Nanjing Medical University, Nanjing, 210009 Jiangsu China; 2 Department of Oncology, The Fourth Affiliated Hospital of Soochow University, Medical Center of Soochow University, Suzhou, 215123 Jiangsu China; 3 Senior Department of General Surgery, The First Medical Center, Chinese PLA General Hospital, Beijing, 100853 China; 4 Institute of Pharmacologic Science, China Pharmaceutical University, Nanjing, 210009 Jiangsu China; 5 Department of Pediatrics, Chui Yang Liu Hospital Affiliated to Tsinghua University, Beijing, 100022 China

**Keywords:** Lung cancer, OTUD3, DR5, Rolapitant, Inhibitor, Apoptosis

## Abstract

**Background:**

Lung cancer is cancer with the highest morbidity and mortality in the world and poses a serious threat to human health. Therefore, discovering new treatments is urgently needed to improve lung cancer prognosis. Small molecule inhibitors targeting the ubiquitin-proteasome system have achieved great success, in which deubiquitinase inhibitors have broad clinical applications. The deubiquitylase OTUD3 was reported to promote lung tumorigenesis by stabilizing oncoprotein GRP78, implying that inhibition of OTUD3 may be a therapeutic strategy for lung cancer.

**Results:**

In this study, we identified a small molecule inhibitor of OTUD3, Rolapitant, by computer-aided virtual screening and biological experimental verification from FDA-approved drugs library. Rolapitant inhibited the proliferation of lung cancer cells by inhibiting deubiquitinating activity of OTUD3. Quantitative proteomic profiling indicated that Rolapitant significantly upregulated the expression of death receptor 5 (DR5). Rolapitant also promoted lung cancer cell apoptosis through upregulating cell surface expression of DR5 and enhanced TRAIL-induced apoptosis. Mechanistically, Rolapitant directly targeted the OTUD3-GRP78 axis to trigger endoplasmic reticulum (ER) stress-C/EBP homologous protein (CHOP)-DR5 signaling, sensitizing lung cancer cells to TRAIL-induced apoptosis. In the vivo assays, Rolapitant suppressed the growth of lung cancer xenografts in immunocompromised mice at suitable dosages without apparent toxicity.

**Conclusion:**

In summary, the present study identifies Rolapitant as a novel inhibitor of deubiquitinase OTUD3 and establishes that the OTUD3-GRP78 axis is a potential therapeutic target for lung cancer.

**Supplementary Information:**

The online version contains supplementary material available at 10.1186/s12964-024-01519-8.

## Introduction

Globally, cancer is a serious threat to human health and is considered to be the main cause of death [1]. The latest data indicate that lung cancer has the highest mortality rate among tumors [1]. Lung cancer can be divided into non-small cell lung cancer (NSCLC) and small-cell lung cancer (SCLC) according to histopathology [2]. Among them, NSCLC accounts for about 85% of all lung cancers [2]. In the past two decades, scientists have made a series of major advances in the treatment of lung cancer, and the use of small molecule tyrosine kinase inhibitors and immunotherapy has helped some patients experience an unprecedented survival benefit [3]. However, there remains a considerable population of lung cancer patients with inherent drug resistance, or who adaptively develop resistance to inhibitors and immunotherapy [4, 5]. Therefore, there is a need for continued research on new drug development and rational drug combination therapy to improve lung cancer prognosis.

Ubiquitination is a highly conserved form of protein post-translational modification, in which ubiquitin E3 ligase and deubiquitinases (DUBs) are the key editors of the ubiquitination process [6]. E3 ligase can transfer ubiquitin (Ub) to substrate proteins, while DUBs cleave and remove the Ub chains from substrate proteins [7]. Many studies have shown that ubiquitination plays an important role in cancer pathogenesis and progression. A larger number of studies demonstrated that targeting ubiquitination has therapeutic potential in multiple cancers [6, 8, 9]. Scientists have developed molecular-targeted drugs that target E3 and DUBs to combat malignant neoplasms [10], especially those targeting *USP1* [11], *USP7* [12, 13], and *OTUB1* [14–16], which display potential for clinical application. For example, the inhibitor ML323 of USP1 can potentiate cisplatin cytotoxicity in NSCLC and osteosarcoma cells [11]. The small molecule inhibitor, P5091, is an inhibitor of USP7 and induces apoptosis in Multiple Myeloma cells [12]. Lanatoside C (LanC) is an inhibitor of OTUB1 and can inhibit Multiple Myeloma tumor growth through disrupting the interaction of OTUB1 and c-Maf [14].

Ovarian tumor domain-containing protein 3 (OTUD3), which is a key OTU (ovarian tumor protease) family deubiquitylase, plays important roles in a variety of cancers. Our previous study indicated that OTUD3 promoted lung tumorigenesis through stabilizing the glucose-regulated protein 78 kDa (GRP78) [2, 17]. GRP78, a major endoplasmic reticulum (ER) chaperone, is primarily localized to the ER and participates in unfolded protein response and ER stress [18]. Rolapitant is a high-affinity NK1 receptor antagonist that was approved by FDA as a treatment for nausea and vomiting caused by chemotherapy [19]. The function of Rolapitant in the tumors has not been studied. In our present study, we identified Rolapitant as an inhibitor of OTUD3 that can also promote ER stress DR5-induced apoptosis of lung cancer cells through disrupting the interaction of OTUD3 with GRP78.

Apoptosis includes the extrinsic and intrinsic apoptotic pathways. Death receptors 4 and 5 (DR4/5) or tumor necrosis factor-related apoptosis-inducing ligand receptors 1 and 2 (TRAIL-R1/R2) play an important role in the extrinsic pathway [20]. TRAIL can bind the death receptors and recruit the Fas-associated protein with death domain (FADD), forming a complex known as death-inducing signaling complex (DISC) that leads to activation of caspase-8 and subsequent downstream caspase-3-dependent apoptosis of the cell [21, 22]. DR5, death receptor 5 (also known as Apo2, TRAIL-R2, TRICK2, or Killer/DR5), belongs to the TNF receptor gene superfamily [22, 23] and is an essential component of the extrinsic apoptosis pathway. The transcription expression of DR5 can be induced by stress-related transcription factors, such as p53, FOXO3a, ATF4, and CHOP [24]. In our present study, we found that Rolapitant can promote the expression of the ER stress-induced transcription factor CHOP and Rolapitant upregulated the transcription expression of DR5 through CHOP.

In conclusion, Rolapitant promotes lung cancer cell apoptosis through upregulating cell surface expression of DR5 and enhancing TRAIL-induced apoptosis. Rolapitant directly targets the OTUD3-GRP78 axis to trigger ER stress-related CHOP-DR5 signaling, sensitizing lung cancer cells to TRAIL-induced apoptosis. Moreover, Rolapitant combined Gefitinib or Afatinib have a good synergistic antitumor effect in vivo. Our findings provide a novel approach for lung cancer treatment and are valuable for clinical application.

## Materials and methods

### Cell lines and drug sources

All cells are purchased from the American Type Culture Collection (ATCC). All cell lines were examined by DAPI DNA staining, and the mycoplasma test was negative. The human NSCLC cell lines H1299, A549, H1650, H460, H1975 or human normal lung epithelial cells BEAS-2B, HBE were cultured in RPMI-1640 medium containing 10% fetal bovine serum (FBS) and antibiotics. The reagents used were as follows: Rolapitant (S5476, Selleck), Estradiol Cypionate (S4046, Selleck), Recombinant Human TRAIL/Apo2L (HY-P7306, MCE), Tunicamycin (HY-A0098, MCE).

### Computational virtual screening

The OTUD3 (PDB ID: 4BOU) and OTUD5 (PDB ID: 3TMP) PDB files were obtained from the Protein Data Bank (http://www.rcsb.org/). The structure of OTUD3 was prepared before molecular docking using AutoDockTools-1.5.7. The GridBox was defined to include S1 Ub binding site. The SDF format files of FDA-approved drugs were converted to PDB format using Open Babel 3.1.1. The converted PDB files were then converted to PDBQT format using a ligand preparation script from AutoDockTools-1.5.7. Then, Autodock Vina 1.1.2 was used for the subsequent molecular docking. The small molecules were ranked based on the affinity energy.

All of the visualization of the structure files was performed using PyMOL Molecular Graphics System, Version 2.6.0a Schrödinger, LLC. Protein–ligand interactions were analyzed using the Protein-Ligand Interaction Profiler web tool.

### Microscale thermophoresis (MST) analysis

NanoTemper Monolith NT.115 instrument (NanoTemper Technologies GmbH) was used to perform the The MST analysis. The protein of OTUD3 was labeled and purified using monolith TMRED Red-NHS 2nd Generation protein labeling kit (NanoTemper Technologies GmbH). Measuring the affinity of OTUD3 with drugs:20 nM of the labeled OTUD3 was mixed with drug prepared in 16 different serial concentrations at RT in 1.05*PBS. The mixtures were then loaded into standard glass capillaries (Monolith NT.115 Capillaries). After blowing evenly, the machine was tested and the Initial Fluorescence Analysis program (LED 20%, medium MST power) was used for Analysis through the MO. Control software (NanoTemper Technologies GmbH). Two to four replicate MST measurements were conducted. Datasets were processed with the MO. Affinity Analysis software (NanoTemper Technologies GmbH).

### Antibodies

Antibodies used were anti-GRP78 (cat. no. 11587–1-AP, 1:3000, Proteintech), anti-PERK (cat. no. 20582–1-AP, 1:1000, Proteintech), anti-IgG (mouse. no. sc2025, 1:400, Santa Cruz Biotechnology), anti-P-PERK (cat. no.YP1055, 1:1000, Immunoway), anti-Ubiquitin (cat. no. sc8017, Santa Cruz Biotechnology), anti-OTUD3 (cat. no. CSBPA719399LA01HU, 1:1000, CUSABIO), anti-GAPDH (mouse. no. ab8245, 1:10000, Abcam), anti-DR5 (cat. no. 8074 s, Cell Signaling Technology), anti-Cleaved-Caspase-8 (cat. no. 9496 s, Cell Signaling Technology), anti-Caspase-8 (cat. no. 4790 s, Cell Signaling Technology), anti-Cleaved-Caspase-3 (cat. no. 9661 s, Cell Signaling Technology), anti-IRE1 (cat. no. 3294; 1:1000; Cells Signaling Technology, Inc.), anti-ATF6 (cat. no. 65880; 1:1000; Cells Signaling Technology, Inc.), anti-ATF4 (cat. no. 11815; 1:1000; Cells Signaling Technology, Inc.), anti-p-eIF2 (cat. no. 5324; 1:1000; Cells Signaling Technology, Inc.), anti-XBP-1 s (cat. no. 12782; 1:1000; Cells Signaling Technology, Inc.), anti-PARP (cat. no. 9532; 1:1000; Cells Signaling Technology, Inc.), anti-CHOP (mouse.no.2895,1:1000, Cells Signaling Technology, Inc.), DR4 (cat.no.42533, 1:1000; Cells Signaling Technology, Inc.), anti-FADD (cat. no. 2782, 1:1000; Cells Signaling Technology, Inc.). The secondary antibodies used were anti-rabbit lgG, HRP-linked Antibody (7074 s), anti-mouse lgG, HRP-linked Antibody (7076 s). All these antibodies were dissolved with 5% skimmed milk in Tris Buffered saline Tween (TBST).

### Colony formation assays

One thousand cells per well of A549, H1299, H460 and H1975 and normal lung epithelial cells of BEAS-2B were planted in a 6-well plate for 24 h. Then cells were treated with different concentrations (0–25 μM) of Rolapitant for 10–14 days until clones could be clearly visible. Cell culture plates were gently washed by PBS twice, fixed with 4% paraformaldehyde phosphate buffer for 15 minutes and then stained with crystal violet for 15 minutes. Washing the excess stain with ddH_2_O and drying them at room temperature for several hours.

### Western blot

Western blot analysis was performed using standard techniques. Lung cancer cells were digested and lysed by RIPA lysis buffer (50 mM Tris-HCl pH 8.0, 150 mM NaCl, 1% NP-40, 0.5% sodium deoxycholate, 0.1% SDS). The ratio of phosphatase inhibitor and protease inhibitor to RIPA lysis buffer was 1:1:100. Proteins were separated by SDS-PAGE and transferred to polyvinylidene difluoride (PVDF) membranes (Merck Millipore, Darmstadt, Germany). These membranes were blocked with 5% skimmed milk for 1 h and then incubate overnight with specific antibody. Washing the membranes three times with Tris Buffered saline Tween (TBST) for 10 minutes each. PVDF membranes were incubated with secondary antibodies for 1 h at room temperature and then washed with TBST four times for 10 minutes each. Finally, Using the ECL Western blotting system (Thermo Scientific, Waltham, MA, USA) to detect the expression of target proteins.

### Cell viability assays

Cell viability assays was performed using the CCK-8 (APEXBIO) test. One thousand cells were seeded in 96-well plates and allowed to grow for 24 h. Then each well was treated with dimethyl sulfoxide (DMSO) or different drug dose of Rolapitant for 0 h, 24 h, 48 h,72 h and 96 h. Next, the medium was removed, and 10 μl of CCK-8 which was dissolved in 90 μl of RPMI 1640 medium (Gibco). The optical density (OD) values of each well were measured at 450 nm using a SpectraMax spectrophotometer (Molecular Devices, San Jose, CA, USA) after being incubated at 37 °C for 1 h.

### siRNA and plasmid transfection

Cells (1× 10^5^ cells/well) were seeded in 6-well plates and allowed to grow to 50% confluent at the time of transfection. Diluting 8 μl of siRNA or 8 μg of plasmid into 200 μl of jetPRIME buffer. Then 4 μl of jetPRIME reagent (Polyplus transfection) was added into the buffer. The buffer was mixed and incubated for 10 minutes at room temperature. Finally, the transfection mix was added into the cells. The sense and anti-sense strands of siRNAs were as follows:

*DR5* siRNA-1 sense:5′-GACCCUUGUGCUCGUUGUCTT-3′ and siRNA-1 antisense: 5′-GACAACGAGCACAAGGGUCTT-3′.

*DR5* siRNA-2 sense: 5′- CCUACAACCUAGAGAAGAATT − 3′ and siRNA-2 antisense: 5′- UUCUUCUCUAGGUUGUAGGTT -3′.

*DR5* siRNA-3 sense: 5′- GAUCAACGUGCUAGAGAAATT − 3′ and siRNA-3 antisense: 5′- UUUCUCUAGCACGUUGAUCTT -3′.

*CHOP* siRNA-1 sense: 5′-CAGUAUCUUGAGUCUAAUATT-3′ and siRNA-1 antisense: 5′-UAUUAGACUCAAGAUACUGTT-3′.

*CHOP* siRNA-2 sense: 5′-GGAGGAAGACCAAGGGAGATT-3′ and siRNA-2 antisense: 5′-UCUCCCUUGGUCUUCCUCCTT-3′.

*CHOP* siRNA-3 sense: 5′-AGGAGAAAGAACAGGAGAATT-3′ and siRNA-3 antisense: 5′-UUCUCCUGUUCUUUCUCCUTT-3′.

The lung cancer cells stably knocking down *OTUD3* were from our previous research [2].

*OTUD3* shRNA-1: 5′-TGGAAATCAGGGCTTAAAT-3′ and *OTUD3* shRNA-2: 5′-GAGTTACACATCGCATATC-3′.

### Immunoprecipitation

A549 and H1299 cells treated with DMSO and Rolapitant respectively were lysed in IP Lysis buffer (50 mM Tris–HCl pH 8.0, 100 mM NaCl, 1 mM EDTA, and 1% Nonidet P40) containing phosphatase inhibitors and protease inhibitors on ice for 30 minutes and then purified via centrifugation for 15 minutes, 12,000 g at 4 °C. The supernatants were incubated with diluted anti-lgG antibody, anti-GRP78 antibody and the magenetic beads (Thermo Fisher Scientific, MA, USA) overnight at 4 °C. The proteins were eluted with 2 × SDS-PAGE Sample Loading Buffer (Beyotime Biotechnology,Jiangsu, China) for 10 minutes at 100 °C after being washed by the lysis buffer. Immunocomplexes were determined by western blot analysis.

### In vivo GRP78 ubiquitylation assay

For in vivo GRP78 ubiquitylation assays, Flag-OTUD3 or Flag-OTUD3^C76A^, Myc-GRP78 and HA-Ub were transfected into HEK293T cells. The different groups were treated with 5 μM of Rolapitant or an equal volume of DMSO for 40 h. Cells were lysed with RIPA lysis buffer after treated with 20 μM of the proteasome inhibitor MG132 (Calbiochem) for 6 h and incubated with anti-Myc antibody for 3 h and protein A/G agarose beads (Santa Cruz) for a further 6 h at 4 °C. Then the beads were washed three times with RIPA buffer. The proteins were released from the beads by boiling in SDS–PAGE sample buffer and analyzed by immunoblotting with anti-HA monoclonal antibody (MBL).

### In vitro deubiquitination assays

Use the purified OTUD3 OTU (aa 52–209) (purchased from SinoBiological) and Di-Ubiquitin (P20023, purchased from Solarbio) for the in vitro deubiquitination assay. Add 2 μg of OTUD3 OTU (aa 52–209) and 2 μg of Di-Ubiquitin to 40 μL buffer (20 mM HEPES, pH 7.2, 150 mM NaCl, and 10 mM DTT). Then, Rolapitant or DMSO were added to the different groups. Carry out the deubiquitination reaction for 3 h at 37 °C with occasional shaking. Stop the in vitro reaction by adding 40 μL of 2 × loading buffer and analyze by western blot.

### Co-immunoprecipitation assays

HEK293T cells were transfected with indicated plasmids using Polyethylenimine (PEI, Invitrogen) reagent according to the manufacturer’s protocol, and treated with DMSO or different concentrations of Rolapitant. For immunoprecipitation assays, cells were lysed with HEPES lysis buffer (20 mM HEPES, pH 7.2, 50 mM NaCl, 0.5% Triton X-100, 1 mM NaF and 1 mM dithiothreitol) supplemented with a protease-inhibitor cocktail (Roche). Immunoprecipitations were performed using the indicated primary antibody and protein A/G agarose beads (Santa Cruz) at 4 °C. The immunocomplexes were then washed with HEPES lysis buffer four times. Both lysates and immunoprecipitates were examined using the indicated primary antibodies followed by detection with the related secondary antibody and the SuperSignal West Pico chemiluminescence substrate (Thermo).

### GST pulldown assays

Bacterially expressed GST and GST-OTUD3 bound to glutathione-Sepharose 4B beads (from GE) were incubated with Myc-GRP78 or Flag-OTUD3 expressed in HEK293T cells for 4 h at 4 °C in the presence of DMSO or different concentrations of Rolapitant. Then the beads were washed with GST binding buffer (100 mM NaCl, 10 mM Tris, 50 mM NaF, 2 mM EDTA, 0.5 mM Na3VO4 and 1% NP40) four times and proteins were eluted, followed by western blotting or Coomassie blue staining.

### Flow cytometry-based apoptosis detection

Flow cytometry-based apoptosis assay was performed using the Annexin V-FITC Apopotosis Detection Kit (KeyGEN BioTECH) according to the manufacturer’s instructions. A549, H1299 and BEAS-2B cells were seeded in 6-well plates at a concentration of 2 × 10^5^ cells/well. Cells were lysed by Trypsin-EDTA (0.25%) (GIBCO) after being treated with different doses of Rolapitant for 24 h. 500 μl of binding buffer with 5 μl of Annexin V-FITC were added into the plate after being washed twice by PBS. Then 5 μl of Propidium iodide (PI) was added after 10 minutes and keeping the cells in the dark. Analysis was performed on a flow cytometer (BD Biosciences) in 1 h and data were processed by Modfit software.

### Flow cytometry-based cell surface DR5 analysis

For flow cytometric analyses, cells were lysed with Trypsin, blocked by PBS, 10% FBS for 10 minutes on ice and subsequently incubated with 5 μl of PE-labeled mouse isotype-mached Control lgG1 (eBioscience, invitrogen) and 5 μl of CD262 (DR5) monoclonal antibody conjugated with PE fluorochrome (eBioscience, invitrogen). All stains were performed using PBS, 10% FBS for 30 minutes in the dark at either RT or 4 °C. Cells were suspended in 400 μl of PBS after two washing steps by PBS and then analyzed on flow cytometer (BD Biosciences). Data was processed and presented using Modfit software.

### RT-qPCR

Total RNA was collected using Takara MiniBEST Universal RNA Extraction Kit (Takara Biotechnology CO., Ltd) according to the manufacturer’s instructions. The concentration and quality of RNA were detected by 260/280 nm absorbance. Using PrimeScript RT Master Mix (Takara biotechnology Co., Ltd) reverse transcripts RNA into high-volume DNA. Quantitative real-time PCR was performed with ABI 7300 PCR system (Applied Biosystems, CA, USA), using SYBR Green Master Mix (Termo Fisher Scientific, USA). All primers were designed and synthesized by Sangon Biotechnology Co. (Shanghai, China), according to the gene sequence in Genbank. Following an initial denaturation at 95 °C for 30 s, 45 cycles of PCR amplification were performed at 95 °C for 5 s and 60 °C for 30 s. Relative expression of target gene mRNA was normalized to GAPDH as an internal control and calculated using 2^−△△^CT. The primer sequences are as following: *GRP78* forward: 5′-CTGTCCAGGCTGGTGTGCTCT-3′ and *GRP78* reverse: 5′-CTTGGTAGGCACCACTGTGTTC-3′; *DR5* forward: 5′-GCCCCACAACAAAAGAGGTC-3′ and *DR5* reverse: 5′-AGGTCATTCCAGTGAGTGCTA-3′; *GAPDH* forward: 5′-ACGGATTTGGTCGTATTGGG-3′ and *GAPDH* reverse: 5′-CGCTCCTGGAAGATGGTGAT-3′;

*CHOP* forward: 5′- AGCCAAAATCAGAGCTGGAA-3′ and *CHOP* reverse: 5′- TGGATCAGTCTGGAAAAGCA-3′.

### Human tumor xenograft models

The animal experimental protocols were approved by the Animal Care and Use Committee of Nanjing Medical University, China. Female BALB/c nude mice (4–6 weeks old) were purchased from Beijing Vital River Laboratory Animal Technology Co., Ltd. Mice were received a 200 μl A549 cells suspensions (2 × 10^7^ cells/mL) by subcutaneous injection under the armpit. Then mice were randomized into three groups when the tumor size reached approximately 150–200 mm^3^. Rolapitant was dissolved in 10% DMSO, 40% PEG300, 5% Tween-80, 45% saline at the final concentration of 100 mg/ml. Eight mice in treatment group were given 75 or 50 mg/kg Rolapitant every 3 days. Control mice were injected with drug vehicle. For combined treatment xenograft models, mice were received a 200 μl A549 cells suspensions (2 × 10^7^ cells/mL) by subcutaneous injection under the armpit. After tumors had reached ~ 100 mm^3^, mice were randomized into the following five groups (*n* = 6): (1) vehicle control (10% DMSO, 40% PEG300, 5% Tween-80, 45% saline); (2) 50 mg/kg Rolapitant; (3) 20 mg/kg Gefitinib; (4) 4 mg/kg Afatinib; (5) 50 mg/kg Rolapitant plus 20 mg/kg Gefitinib; (6) 50 mg/kg Rolapitant plus 4 mg/kg Afatinib. Gefitinib and Afatinib were administered by intraperitoneal injection three times a week for 3 weeks. The experiment was complete when the tumor size of the control group reached 2000 mm^3^**.** The weight and tumor size of the mice were monitored every 3 days. Tumor tissues were collected immediately and detected when the mice were killed.

## Statistical analysis

All experiments were performed independently at least three times and in triplicate each time. Data were analyzed with GraphPad Prism 8.0. All Statistical analysis were calculated by Student’s *t*-test or the analysis of variance (ANOVA). *P*-values < 0.05 were considered statistically significant.

## Results

### Identification of small-molecule OTUD3 inhibitors

**Fig. 1:**
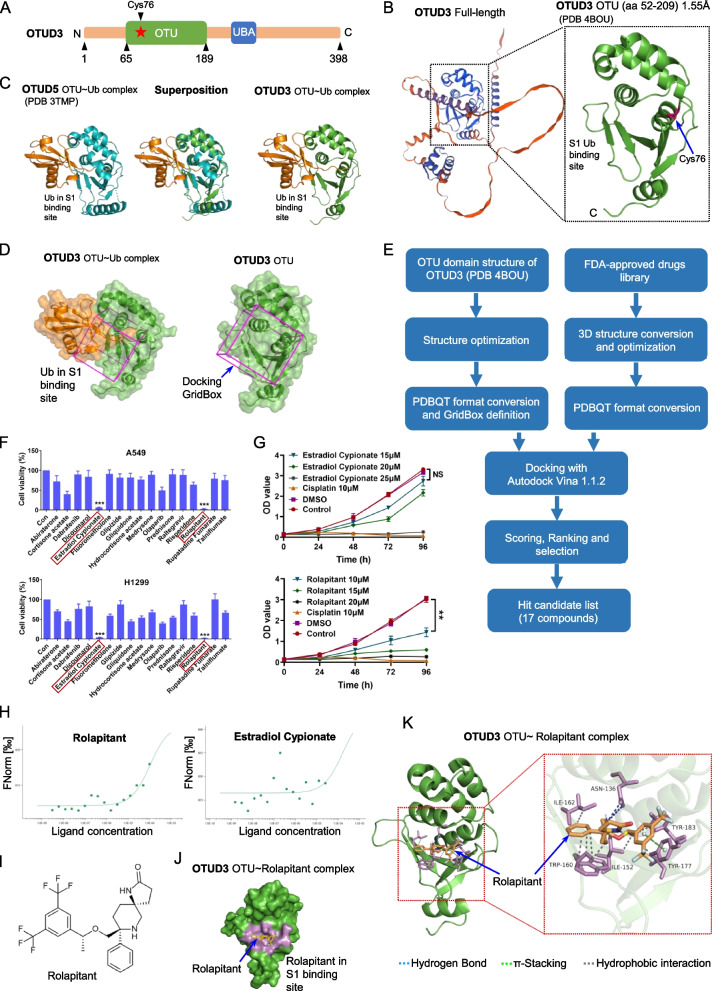
Identification of small-molecule OTUD3 inhibitor. **A** Diagram of human OTUD3, showing ovarian tumor (OTU) and ubiquitin-associated (UBA) domains, and active site (Cys76) directly involved in catalysis. **B** Crystal structure of the OTUD3 OTU domain. A cartoon representation is shown. The S1 Ub-binding site, Cys76, N termini, and C termini are labeled. **C** Left: Crystal structure of the OTUD5 OTU domain (green) bound to Ub (orange) (PDB-ID:3TMP). Middle: Structure of the OTUD3 OTU domain (blue) superimposed to the OTUD5 OTU domain (green) bound to Ub (orange). Right: Approximate structure of the OTUD3 OTU domain (blue) bound to Ub (orange), Ub position was determined by sequence and structural alignment with 3TMP. **D** Identification of the docking GridBox that encloses the S1 Ub-binding site. **E** Schematic diagram of computational virtual screening based on molecular docking. **F** A549 and H1299 treated with different candidate drugs at the indicated concentration (50 μM) for 72 h and cell survival rate was detected by CCK-8 assays. **G** A549 cells treated with DMSO, Estradiol Cypionate and Rolapitant at the indicated concentrations (15–25 μM) for 0–96 h. Cisplatin (10 μM) was used as a positive control. **F**,** G** The results were presented as the mean ± SD from 3 independent experiments. (*n* = 3; *, *p* < 0.05; **, *p* < 0.01, ***, *p* < 0.001 vs. DMSO). **H** MST (microscale thermophoresis) assays were uesd to detect the interaction of OTUD3 with Rolapitant and Estradiol Cypionate in vitro. **I** Chemical structure of Rolapitant. **J** Surface representation of the structure of the OTUD3 OTU domain in complex with Rolapitant. The residues around Rolapitant are highlighted in violet. **K** Overall structure of OTUD3 OTU domain in complex with Rolapitant, and Close-up view of the compound binding site highlighting key residues. OTUD3: green cartoon; Rolapitant: orange sticks; OTUD3 (Asn136, Ile152, Trp160, Ile162, Tyr177 and Tyr183) side chains are represented as sticks. Dashed lines showing the key interactions between OTUD3 and Rolapitant. Blue dashed lines represent hydrogen bonds. Green dashed lines indicate π-stacking interactions. Grey dashed lines indicate hydrophobic interactions. Binding site detail, showing the interactions between Rolapitant and OTU domain residues. Hydrogen bonds are formed between Asn136 and Rolapitant. π-stacking interactions are formed between Tyr177 and Rolapitant. Hydrophobic interactions are formed between Ile152, Trp160, Ile162, Tyr177, Tyr183 and Rolapitant. The PLIP package was used to analyze protein-ligand interactions. **B**,** C**,** D**, **J**,and **K** The images were generated with The PyMOL Molecular Graphics System (version 2.6.0a0)

OTUD3 contains an ovarian tumor (OTU) domain and a ubiquitin-associated (UBA) domain (Fig. 1A). The OTU domain is the catalytic domain and is essential for OTUD3 to perform deubiquitinating activity and interact with substrates [25, 26]. The OTUD3 (PDB: 4BOU) PDB file was downloaded from the Protein Data Bank (http://www.rcsb.org/) (Fig. 1B). All of the heterogeneous atoms were removed, and the 4BOU chain A was selected for subsequent molecular docking. The S1 Ub binding site is critical for the deubiquitinating activity of OTUD3, which is the most frequent binding site for DUB inhibitors with the most potent and specific binders reported to date [27, 28]. Therefore, the S1 Ub binding site was determined as the ligand binding site. However, the structure of OTUD3 in complex with Ub was not yet resolved. Considering that the structure of OTUD3 is highly similar to OTUD5 with a low root mean square deviation (~ 0.8 Å), we performed superimposition of the OTUD3 structure with the structure of OTUD5 OTU-Ub complex to identify the Ub-binding site of OTUD3 (Fig. 1C). Then, the docking grid box was set to enclose the entire S1 Ub-binding site and all the amino acid residues around it (Fig. 1D). Autodock Vina 1.1.2 was used for the subsequent molecular docking [29]. FDA-approved drug library was screened, and 17 candidate hits with the highest docking scores were identified for further biological experimental verification (Fig. 1E). Our previous study indicated that deubiquitinase OTUD3 promoted the progression of lung cancer through stabilizing GRP78 [2]. Therefore, cell proliferation assay was chosen as a further screening strategy for the OTUD3 inhibitors. A549 and H1299 cells were incubated with 50 μM of each compound, and their survival rate was detected by Cell Counting Kit 8 (CCK-8) assay. We found that compounds Estradiol Cypionate and Rolapitant can significantly inhibit the proliferation of A549 and H1299 cells (Fig. 1F). Rolapitant significantly inhibited the proliferation of lung cancer cells at lower concentrations (Fig. 1G). Moreover, MST (microscale thermophoresis) assays shown that OTUD3 could directly bind to Rolapitant in vitro, but not bind well to Estradiol Cypionate (Fig. 1H). Therefore, we chose Rolapitant for further study (Fig. 1I).

We next performed molecular docking of Rolapitant. Molecular docking analysis showed that the affinity energy of Rolapitant to OTUD3 was − 7.4 kcal/mol, and Rolapitant was docked into the S1 Ub-binding site pocket (Fig. 1J). The predicted protein-ligand interactions mode showed that Rolapitant formed multiple interactions with OTUD3, including hydrophobic interactions with Ile152, Trp160, Ile162, Tyr177 and Tyr183, hydrogen bonds with Asn136, and π-stacking bonds with Tyr177(Fig. 1K). Collectively, we identified Rolapitant as an inhibitor of OTUD3 using virtual screening, cell proliferation assays and MST assays, which represented a potentially tractable starting point suitable for further investigation.

### Rolapitant inhibits lung cancer cell growth by targeting OTUD3

**Fig. 2:**
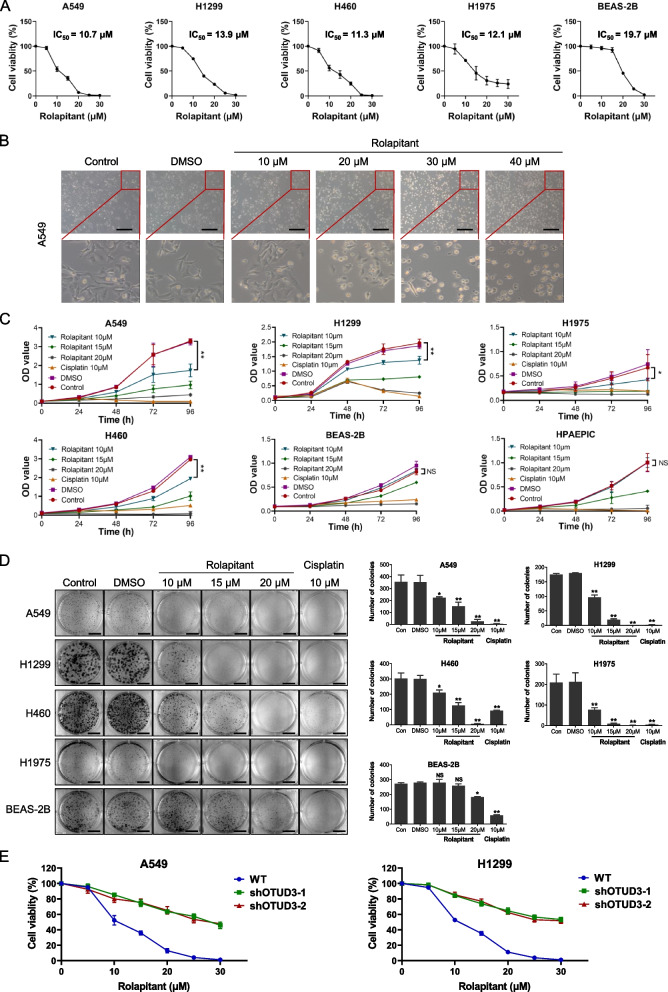
Rolapitant inhibits lung cancer cell growth by targeting OTUD3. **A** A549, H1299, H1975, H460 and BEAS-2B cells were treated with the indicated concentrations (0–40 μM) of Rolapitant for 72 h. Cell viability analysis was determined by CCK-8 assays. IC_50_ was analyzed by nonlinear regression using GraphPad Prism software. **B** Electron microscopy photographs of A549 cells treated with the indicated concentrations (10–40 μM) of Rolapitant or DMSO for 24 h. Scale bar, 100 μm. **C** OD value (450 nm) was determined by CCK-8 assays. A549, H1299, H1975, H460, BEAS-2B and HPAEPIC cells treated with DMSO or the indicated concentrations (10, 15, 20 μM) of Rolapitant at the times indicated. Cisplatin (10 μM) treated cells were used as a positive control. **D** Clone formation analysis of A549, H1299, H1975, H460 and BEAS-2B cells treated with DMSO or the indicated concentrations (10, 15, 20 μM) of Rolapitant for 10–14 days. Scale bar: 1 cm. Bar graph showed the numbers of clone formation. **E** Cell viability analysis of A549, H1299 control and *OTUD3* knocking down groups were determined by CCK-8 assays at the indicated concentration (0–30 μM) of Rolapitant for 72 h. All the results were presented as the mean ± SD from 3 independent experiments. (*n* = 3, *p < 0.05, **p < 0.01, ***p < 0.001, ns = no significance, vs. DMSO)

From preliminary screening, we found that Rolapitant inhibited lung cancer cells at a concentration of 50 μM. Next, we used CCK-8 assays to determine the 50% inhibitory concentration (IC_50_) of Rolapitant. The results showed that the IC_50_ value of Rolapitant in A549, H1299, H460, H1975, and BEAS-2B cells was 10.70, 13.90, 11.30, 12.05, and 19.69 μM, respectively, and the human normal lung epithelial cells had a higher IC_50_ value than lung cancer cells (Fig. 2A). Microscopic images of the cell morphology showed that Rolapitant induced cell death in A549 cells (Fig. 2B). Next, we detected the effects of Rolapitant on human normal lung epithelial cells BEAS-2B, HPAEPIC, and lung cancer cell lines A549, H1299, H1975, and H460. The cells were treated with different concentrations of Rolapitant and 10 μM Cisplatin for 24 h, 48 h, 72 h, and 96 h. The following CCK-8 assays showed that Rolapitant significantly reduced lung cancer cell viability within 10 μM for 48 h, 72 h, 96 h and had no effect on the proliferation of the human normal lung epithelial cells in 10 μM (Fig. 2C). Moreover, colony formation experiments evaluated the effects of Rolapitant on lung cancer cells and BEAS-2B. The results showed that Rolapitant significantly suppressed the colony formation of lung cancer cells in a concentration-dependent manner, but had weaker effects on the proliferation of BEAS-2B cells (Fig. 2D). In addition, when *OTUD3* was knocked down, the growth of lung cancer cells was less affected by Rolapitant (Fig. 2E). These results implied that Rolapitant suppresses the proliferation of lung cancer cells via OTUD3.

### Rolapitant inhibits the deubiquitinating activity of OTUD3

**Fig. 3:**
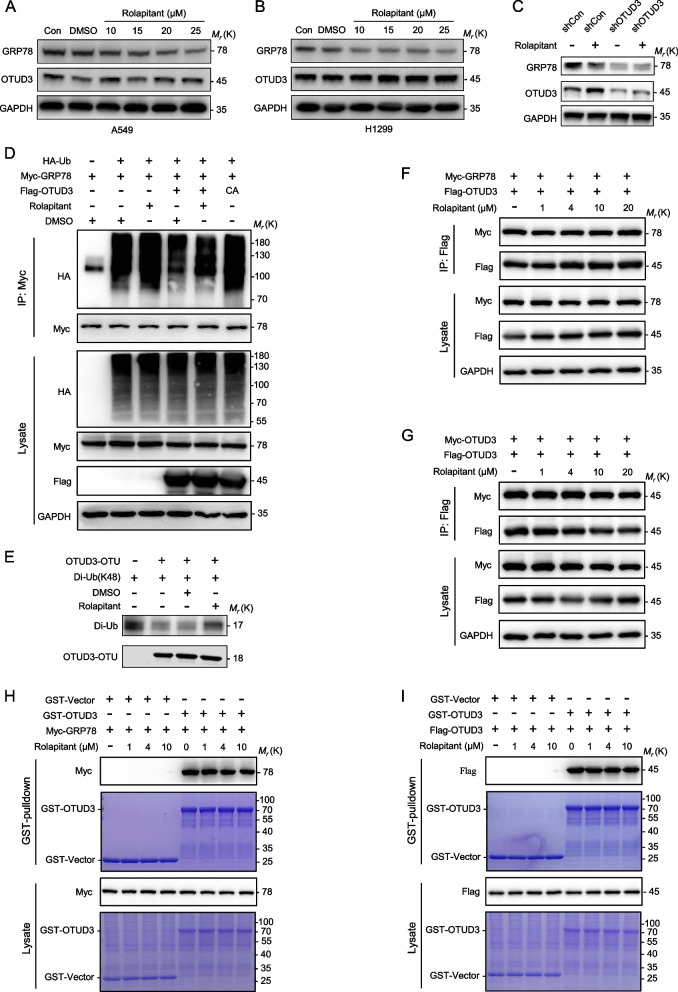
Rolapitant inhibits the deubiquitinating activity of OTUD3. **A**,** B** Western blot analysis of GRP78 and OTUD3 levels in A549 and H1299 cells treated with DMSO or indicated concentrations (0–25 μM) of Rolapitant for 48 h. GAPDH was used as a loading control. **C** *OTUD3* was knocked down by shRNA in H1299 cells. H1299 cells were treated with 10 μM Rolapitant for 48 h, and cell lysates were assessed by western blot. **D** HEK293T cells were transfected with HA-Ub, Myc-GRP78, Flag-OTUD3, Flag-OTUD3^C76A^ alone or in combination and incubated with or without 5 μM Rolapitant for 46 h. Proteasome inhibitors (20 μM MG132) were added 6 h before lysis. Cell lysates were immunoprecipitated with anti-Myc antibody, followed by western blot with indicated antibodies. **E** OTUD3 OTU (aa 52–209) and K48-linked Di-Ubiquitin were incubated with 10 μM Rolapitant for 3 h at 37 °C, followed by western blot with indicated antibodies. **F** Flag-OTUD3 and Myc-GRP78 were co-transfected into HEK293T cells with indicated concentrations of Rolapitant for 24 h. Cell lysates were subjected to immunoprecipitation with anti-Flag antibody, followed by western blot with indicated antibodies. **G** Flag-OTUD3 and Myc-OTUD3 were co-transfected into HEK293T cells with indicated concentrations of Rolapitant for 24 h. Cell lysates were subjected to immunoprecipitation with anti-Flag antibody, followed by western blot with indicated antibodies. **H** HEK293T cells transfected with Myc-GRP78 were lysed and lysates incubated with GST or GST-OTUD3 proteins with increasing concentrations of Rolapitant treatment for 4 h. Proteins retained on Sepharose were blotted with the indicated antibodies, or visualized by SDS-PAGE and Coomassie blue staining. **I** Purified GST-OTUD3 and GST proteins were incubated with extracts from Flag-OTUD3 transfected HEK293T cells with increasing concentrations of Rolapitant treatment for 4 h. Proteins retained on Sepharose were blotted with the indicated antibodies, or visualized by SDS-PAGE and Coomassie blue staining. All panels are representative results of three or more independent experiments

Our previous study confirmed that OTUD3 promotes lung tumorigenesis by stabilizing the substrate GRP78 [2]. Therefore, we examined the effect of Rolapitant on the protein level of GRP78. A549 and H1299 cells were treated with different concentrations of Rolapitant (10, 15, 20, 25 μM), followed by western blot to detect the protein level of GRP78 and OTUD3. The results showed that Rolapitant markedly decreased the protein level of GRP78 compared with the blank control and DMSO solvent groups, but had no effect on OTUD3 (Fig. 3A, B). In addition, when *OTUD3* was knocked down, the effect of Rolapitant on reducing the protein level of GRP78 was significantly decreased (Fig. 3C). This result provided further evidence that the effect of Rolapitant depends on OTUD3.

To verify whether the downregulation effect of Rolapitant on GRP78 resulted from inhibition of the deubiquitinating activity of OTUD3, a GRP78 ubiquitylation assay was performed. Flag-OTUD3 WT (wild-type) or Flag-OTUD3 C76A, Myc-GRP78 and HA-Ub were transfected into HEK293T cells as indicated. The different groups were treated with 5 μM of Rolapitant or an equal volume of DMSO for 46 h. A GRP78 ubiquitylation assay showed that overexpression of OTUD3 WT significantly reduced the level of GRP78 ubiquitination, and the catalytically inactive mutant C76A did not reduce the level of GRP78 ubiquitination (Fig. 3D). However, when OTUD3 WT overexpressing cells were treated with Rolapitant, GRP78 ubiquitination was increased, reversing the deubiquitination of OTUD3 (Fig. 3D).

Next, we performed in vitro deubiquitination assays to further verify whether Rolapitant inhibits the deubiquitinating activity of OTUD3 in vitro. The results indicated that Rolapitant inhibited the deubiquitinating activity of OTUD3 to cleave K48- linked di-Ub (Fig. 3E).

Our previous study showed that the N-terminal OTU domain of OTUD3 mediated the physical interaction with GRP78 [2]. Given that Rolapitant binds to the OTU domain, we further investigated whether Rolapitant disrupted the interaction between OTUD3 and GRP78 by performing co-IP and GST pull-down assays. Co-IP and GST pull-down assays confirmed that Rolapitant did not affect the interaction between OTUD3 and GRP78 (Fig. 3F, H). In addition, OTUD3 formed homodimers to perform deubiquitinating activity [25]. Co-IP and GST pull-down assays suggested that Rolapitant also did not affect the dimerization of OTUD3 (Fig. 3G, I).

Taken together, Rolapitant enhanced degradation of GRP78 by inhibiting the deubiquitinating activity of OTUD3 and increasing GRP78 ubiquitylation. These results are consistent with the expected mechanism of action of an OTUD3 inhibitor.

### Rolapitant promotes apoptosis of lung cancer cells by upregulating DR5

**Fig. 4:**
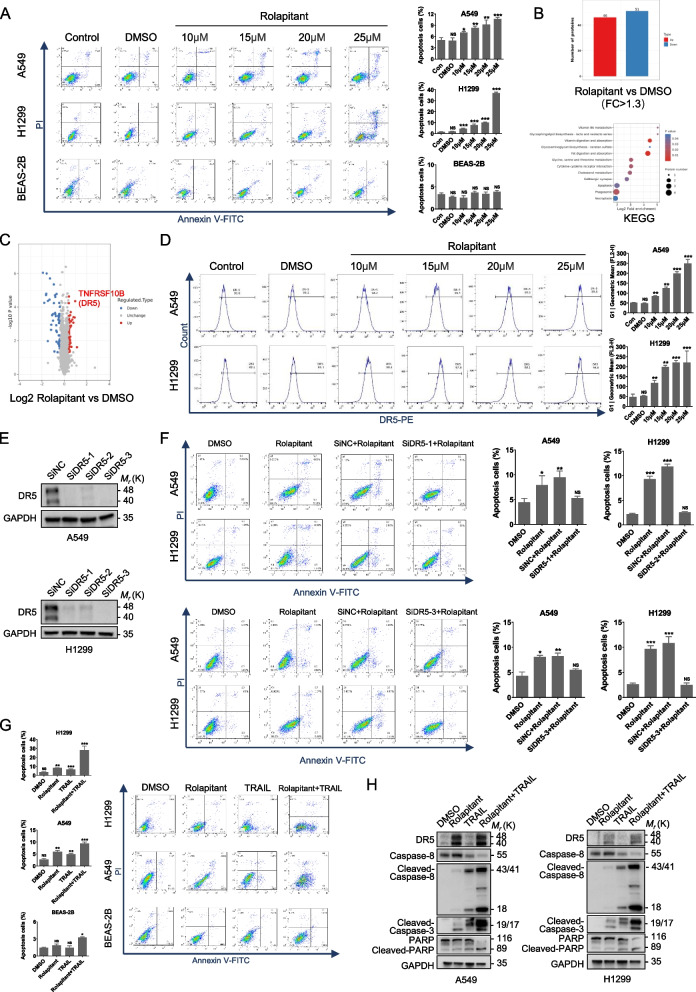
Rolapitant promotes apoptosis of lung cancer cells by up-regulating DR5. **A** Flow cytometric analysis of Annexin V-FITC/PI staining in A549, H1299 and BEAS-2B cells treated with DMSO or Rolapitant (10, 15, 20, or 25 μM) for 48 h. Bar graph showed the percentage of apoptosis. **B**,** C** Proteomic analysis showed the up-regulated and down-regulated proteins in A549 treated with DMSO or Rolapitant (10 μM). Bubble plots showed the pathway of KEGG enrichment. Volcano plots illustrated the differentially expressed proteins in A549 treated with DMSO or Rolapitant (10 μM). **D** Flow cytometric analysis showed the different surface expression levels of DR5 receptor in A549 and H1299 treated with DMSO or the indicated concentrations (10, 15, 20, or 25 μM). Bar graph showed the mean fluorescence intensity of DR5 receptors on the membrane. **E** Western blot analysis of the three independent *DR5* siRNA knockdown effects. **F** DR5 siRNA knocking down and control A549 and H1299 cells treated with DMSO or Rolapitant (10 μM) for 48 h were analyzed by Flow cytometric. Bar graph showed the percentage of apoptosis. **G** A549, H1299 and BEAS-2B cells were treated with DMSO or Rolapitant (10 μM) for 22 h and then exposed to TRAIL (100 ng/ml) for 2 h. Flow cytometric analysis of Annexin V-FITC/PI staining was used to detect the apoptosis cells. Bar graph showed the percentage of apoptosis. **H** Western blot analysis of DR5, Cleaved-Caspase-8, Cleaved-Caspase-3, PARP and Cleaved-PARPP levels in A549 and H1299 cells treated with DMSO or Rolapitant (10 μM) in the absence or presence of TRAIL (100 μg/ml). All the results were presented as the mean ± SD from 3 independent experiments. (*n* = 3, *p < 0.05, **p < 0.01, ***p < 0.001, ns = no significance, vs. DMSO)

To find out whether Rolapitant induced lung cancer cells apoptosis, lung cancer cells treated with different concentrations Rolapitant were subjected to flow cytometry (Fig. 4A). The results revealed that Rolapitant induced marked apoptosis of A549 and H1299 cells but had no effect on the apoptosis of BEAS-2B cells (Fig. 4A). As Rolapitant can induce apoptosis in lung cancer cells, we performed proteome sequencing to further explore its mechanism for apoptosis. Consistently, the results of proteome sequencing indicated that the apoptosis was one of the most important enrichment pathways (Fig. 4B). The analysis of proteome sequencing showed that DR5 may play an important role in modulating apoptosis upon using Rolapitant (Fig. 4C). To investigate whether Rolapitant impacts the level of DR5, we employed flow cytometry in A549 and H1299 cells treated with different concentrations of Rolapitant. Flow cytometric analysis showed that the cell surface expression of DR5 was raised following increasing concentrations of Rolapitant (Fig. 4D). Figure 4E has shown that the three independent *DR5* siRNAs all showed significant effects. We then established A549 and H1299 cells with knockdown of *DR5* by interfering small RNA (si*DR5*–1 and si*DR5*–3) to verify whether Rolapitant triggered apoptosis through DR5. Flow cytometric analysis illustrated that knocking down *DR5* significantly decreased the percentage of apoptosis in A549 and H1299 cells treated with Rolapitant compared to control siRNA (Fig. 4F). Consistently, there was little change between control siRNA in the presence of Rolapitant and Rolapitant alone.

Previous studies have elucidated that TRAIL is a member of TNF superfamily, which can activate the apoptosis pathway by binding to related death receptor DR4 or DR5 in cancer cells [30]. Hence, we further tested the percentage of apoptosis in A549 and H1299 treated with Rolapitant in the presence or absence of TRAIL. Flow cytometric analysis showed that the combination of Rolapitant and TRAIL dramatically increased the rate of apoptosis, compared to Rolapitant or TRAIL alone (Fig. 4G). However, there was no change in normal lung epithelial cells (BEAS-2B) with either Rolapitant alone, TRAIL, or a combination of the two drugs (Fig. 4G). In addition, apoptosis was significantly induced by the co-treatment of Rolapitant/TRAIL, as indicated by cleaved-caspase-8, cleaved-PARP, and cleaved-caspase-3 through upregulating DR5 (Fig. 4H). It is worthwhile to note that there was no significance between DMSO and TRAIL alone, which is consistent with the clinical observation that patients can easily acquire resistance to TRAIL [31].

In conclusion, Rolapitant facilitated the apoptosis of human lung cancer cells by upregulating DR5 and had little impact on normal human lung epithelial cells. Moreover, Rolapitant may have the potential to reverse TRAIL resistance in clinical settings.

### Rolapitant facilitates activation of the DR5 signaling pathway and apoptosis of lung cancer cells by upregulating CHOP

**Fig. 5:**
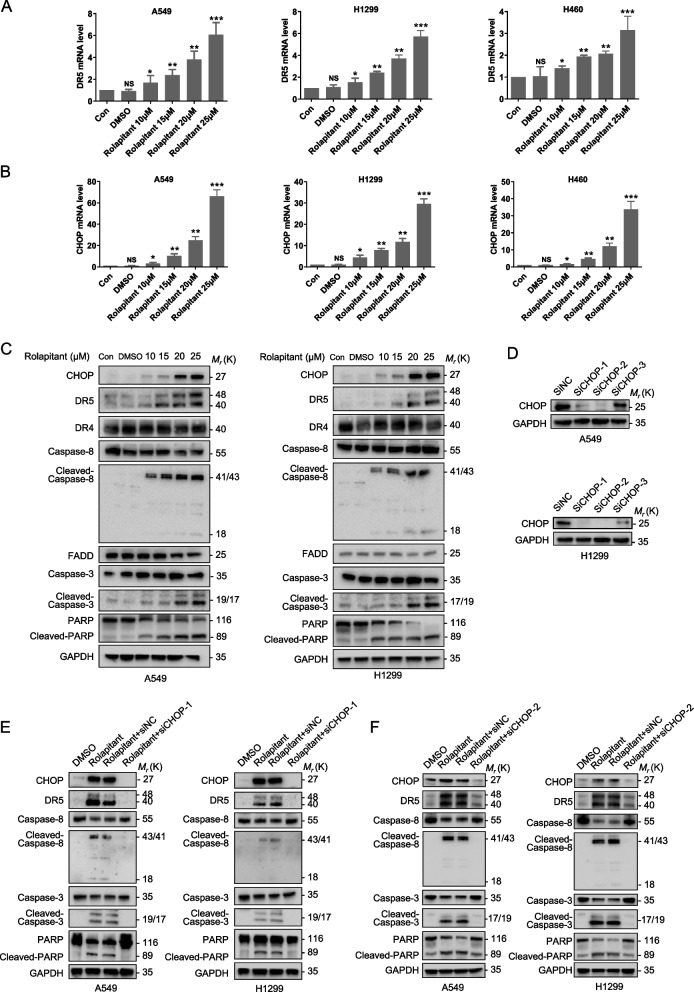
Rolapitant facilitated the activation of DR5 signaling pathway and apoptosis of lung cancer cells by up regulating CHOP. **A**,** B** *DR5* and *CHOP* expression was confirmed in A549, H1299 and H460 cells treated with DMSO or indicated concentrations (10, 15, 20, or 25 μM) of Rolapitant for 48 h by qRT-PCR. **C** Western blot analysis of CHOP, DR5, DR4, Caspase-8, Cleaved-Caspase-8, Caspase-3, Cleaved-Caspase-3 and FADD levels in A549 and H1299 cells treated with DMSO or indicated concentrations (10, 15, 20, or 25 μM) of Rolapitant for 48 h. GAPDH was used as a loading control. **D** Western blot analysis of the three independent *CHOP* siRNA knockdown effects. **E**, **F** *CHOP* siRNA knocking down and control A549 and H1299 cells were treated with DMSO or Rolapitant (10 μM) for 48 h. The expression of Caspase-8, Cleaved-Caspase-8, Caspase-3, Cleaved-Caspase-3, PARP and Cleaved-PARP were determined by western blot

The results above indicated that Rolapitant induced apoptosis via upregulating DR5. Consequently, we further investigated the upstream regulator of DR5. Published studies suggest that the apoptosis of cancer cells can be induced by the CHOP-DR5 axis [32]. Thus, we hypothesized that Rolapitant could promote DR5 signaling pathway by upregulating CHOP. First, we detected the transcriptional level of *DR5* and *CHOP* by qRT-PCR. The transcriptional expression of *DR5* and *CHOP* was dramatically increased with increasing concentrations of Rolapitant (Fig. 5A, B). A previous study revealed that transcription factor CHOP can upregulate the transcriptional expression of *DR5* [33]. Then, we tested the expression of CHOP, DRs, and apoptosis-associated proteins. Western blot analysis showed that CHOP, DR5, and apoptosis-associated protein levels in A549 and H1299 cells were dramatically increased with increasing concentrations of Rolapitant (Fig. 5C). However, the expression of DR4 remained basically unchanged (Fig. 5C). Figure 5D has shown that the two of three independent *CHOP* siRNAs (si*CHOP*-1 and si*CHOP*-2) showed significant effects. Next, we established A549 and H1299 cells with knockdown of *CHOP* by siRNA. The western blot analysis illustrated that the levels of CHOP, DR5, and apoptosis-associated proteins were significantly reduced by *CHOP* siRNA in the presence of Rolapitant compared to the Rolapitant alone (Fig. 5E, F). Together, these data indicate that Rolapitant positively modulated DR5 by upregulating CHOP.

### Rolapitant upregulates CHOP by triggering ER stress through modulating the OTUD3-GRP78 axis in lung cancer cells

**Fig. 6:**
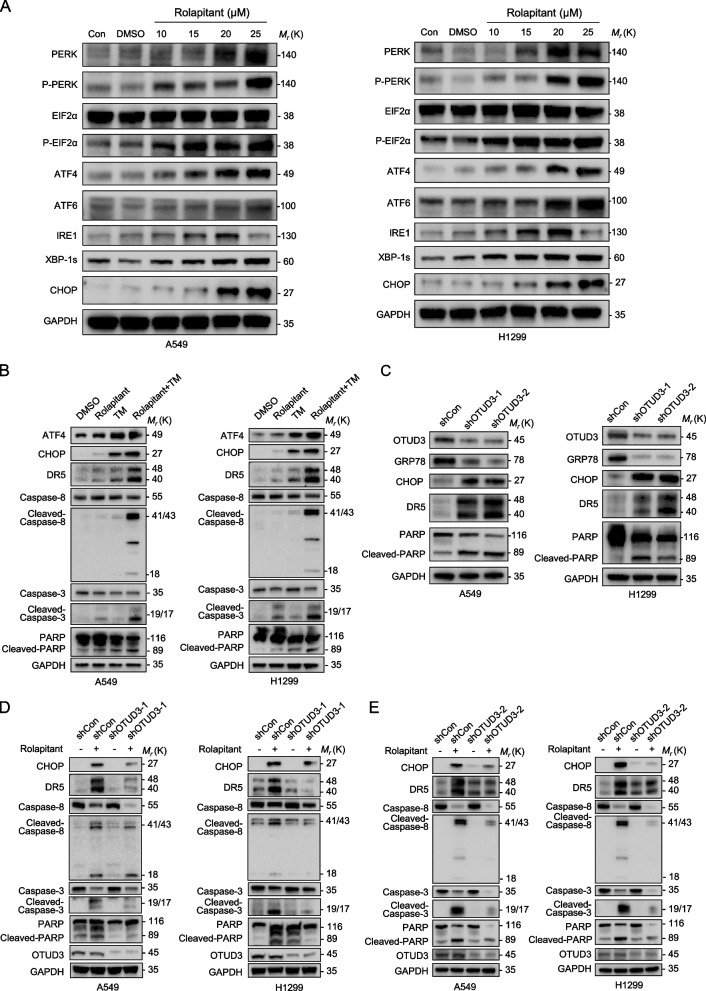
Rolapitant triggered ER stress through down-regulating GRP78 and upregulated CHOP-DR5 pathway. **A** Western blot analysis of PERK, p-PERK, EIF2α, p-EIF2α, ATF4, ATF6, IRE1, XBP-1 s and CHOP levels in A549 and H1299 cells treated with DMSO or indicated concentrations (10, 15, 20, or 25 μM) of Rolapitant for 48 h. GAPDH was used as a loading control. **B** Western blot analysis of ATF4, CHOP, DR5, Caspase-8, Cleaved-Caspase-8, Caspase-3, Cleaved-Caspase-3, PARP and Cleaved-PARP levels in A549 and H1299 cells treated with DMSO or Rolapitant (10 μM) in the absence or presence of TM (1 μg/ml) for 24 h. **C** Western blot analysis of GRP78, CHOP, DR5, PARP, Cleaved-PARP and OTUD3 in *OTUD3* shRNA knocking down and control A549 and H1299 cells. GAPDH was used as a loading control. **D**,** E** Western blot analysis of CHOP, DR5, Caspase-8, Cleaved-Caspase-8, Caspase-3, Cleaved-Caspase-3, PARP, Cleaved-PARP and OTUD3 in *OTUD3* shRNA knocking down and control A549 and H1299 cells treated with or without Rolapitant (10 μM) for 48 h. GAPDH was used as a loading control

The ER is a peculiar structure where a number of important substances in the cell, such as proteins, lipids, and sugars, are synthesized, in addition to nucleic acids [34, 35]. ER homeostasis is therefore crucial to maintaining the biological functions of the eukaryotic cell [34]. Any changes in the extracellular can trigger the accumulation of misfolded proteins in the ER, which then causes stress and activates the unfolded protein response (UPR) [34]. Any physiological or pathological stimulation can cause ER stress. The ER stress sensor pathway, containing IRE1/sXBP1, PERK/EIF2, and ATF6, regulates major functions to ensure ER homeostasis [34]. Previously, it was discovered that ER stress can initiate apoptosis induced by CHOP and DR5 [36, 37]. Accordingly, we hypothesized that Rolapitant could trigger ER stress, which initiates apoptosis induced by the CHOP-DR5 axis. Western blot analysis showed that the levels of PERK, p-PERK, p-EIF2α, ATF4, ATF6, IRE1, and XBP1S in A549 and H1299 cells were significantly increased with the increase of Rolapitant concentration (Fig. 6A). At the same time, the expression of EIF2α did not change. These results indicate that Rolapitant could trigger ER stress through influencing all three ER stress sensor pathways. We detected the expression of ATF4, CHOP, DR5, Caspase-3, Cleaved-Caspase-3, Cleaved-Caspase-8, Caspase-8, PARP, and Cleaved-PARP in A549 and H1299 cells treated with Rolapitant in the presence or absence of Tunicamycin (TM), an ER stress inducer (Fig. 6B). Western blot analysis showed that the levels of apoptosis-related proteins were remarkably increased when combined with TM compared to the single drug Rolapitant or TM, indicating that Rolapitant triggers apoptosis through ER stress (Fig. 6B).

Previous studies have indicated that GRP78 can bind and inactivate all three ER stress transducers to regulate the UPR [38]. GRP78 releases the UPR sensors and leads to the activation of the UPR pathways with GRP78 binding to misfolded proteins in the ER [38]. On the contrary, the UPR can be spontaneously triggered with GRP78 depleted or inactivated [38]. Therefore, the protein level of GRP78 is significant for ER homeostasis. Rolapitant can downregulate the GRP78 through inhibiting the deubiquitinating activity of OTUD3. Based on this finding, we investigated whether Rolapitant can trigger ER stress-induced apoptosis through the OTUD3-GRP78 axis. The expressions of CHOP, DR5, and apoptosis-associated proteins were remarkably enhanced in *OTUD3* shRNA-1 or shRNA-2 compared with the control shRNA (Fig. 6C). Moreover, the western blot analysis showed that the levels of CHOP, DR5, and apoptosis-associated proteins were less increased in the presence of Rolapitant when *OTUD3* was knocked down (Fig. 6D, E). All these results suggest that Rolapitant activates the ER stress-CHOP-DR5 pathway through modulating the OTUD3-GRP78 axis.

### Rolapitant inhibits lung cancer cell growth in vivo

**Fig. 7:**
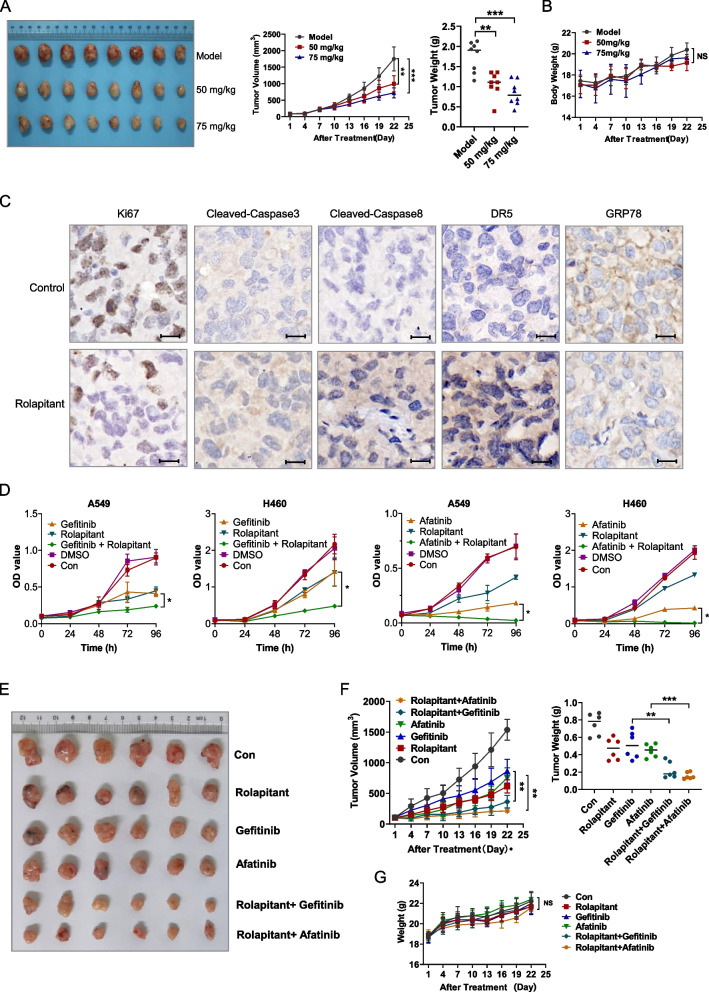
Rolapitant inhibits lung cancer cell growth in vivo. **A** Twenty-four nude mice with visible tumors were randomly assigned to 3 groups: control, Rolapitant (50 mg/kg) and Rolapitant (75 mg/kg). Pictures showed the tumors of all the 24 cases both in control and treatment groups. Tumor volume and weight were measured every 3 days for up to 22 days. **B** Body weight was recorded every 3 days for up to 22 days. **C** Immunohistochemistry illustrated the level of Ki67, Cleaved-Caspase-8, Cleaved-Caspase-3, DR5 and GRP78 in tumor issues containing model and treatment groups. Scale bar: 15 μm. **D** A549 and H460 cells were treated with DMSO, Rolapitant (10 μM), Afatinib (5 μM), Gefifinib (5 μM), Rolapitant plus Afatinib or Rolapitant plus Gefifinib and then assessed for OD value (450 nm) using CCK-8 assays. **E** Thirty-six nude mice with visible tumors were randomly assigned to 6 groups: control, 50 mg/kg Rolapitant, 20 mg/kg Gefitinib, 4 mg/kg Afatinib, 50 mg/kg Rolapitant plus 20 mg/kg Gefitinib, 50 mg/kg Rolapitant plus 4 mg/kg Afatinib. Pictures showed the tumors of all the 36 cases both in control, treatment groups and combined treatment groups. **F** Tumor volume and weight were measured every 3 days for up to 22 days. **G** Body weight was recorded every 3 days for up to 22 days. All the results were presented as the mean ± SD. (*p < 0.05, **p < 0.01, ***p < 0.001, ns = no significance, vs. Control)

As expected, Rolapitant could significantly suppress the proliferation of human lung cancer cells in vitro. To further verify the effects in vivo, 24 nude mice bearing A549 tumors were randomly assigned into three groups: treatment groups that were injected with Rolapitant at 75 mg/kg or 50 mg/kg via gavage, and the control group. Tumor volume and weight were dramatically increased in control mice and were significantly decreased in treatment groups, especially in the 75 mg/kg group (Fig. 7A). The observation of tumors was consistent with the data of tumor volume and weight, indicating that Rolapitant has a tumor-suppressive activity in human lung cancer cells (Fig. 7A). There was no significant difference in average body weight between the control and treatment groups (Fig. 7B). Immunohistochemical results showed that the level of Ki67 and GRP78 was significantly decreased in the treatment group compared to the control (Fig. 7C). In addition, the expressions of Cleaved-Caspase-3, Cleaved-Caspase-8, and DR5 were remarkably increased in the treatment group compared to the control (Fig. 7C).

Afatinib is the first irreversible blocker of the ErbB family, and Gefitinib is a reversible EGFR tyrosine kinase inhibitor (TKI) [39]. Both are approved for the first-line treatment of EGFR mutation-positive NSCLC and later settings [40]. Afatinib and Gefitinib have significantly improved progression-free survival and response rates [41]. However, drug-related adverse events, such as diarrhea, acneiform skin rash, and paronychia, inevitably occurred and the disease unavoidably progressed in recent years [41]. Approximately 60% of patients acquired therapeutic resistance to the EGFR TKIs [39]. The discovery of new drugs and combinations of treatments is therefore urgently needed. Hence, we detected the effects of combination treatments containing Afatinib and Gefitinib in the presence or absence of Rolapitant. The results provide powerful evidence that combined treatments induce greater reduction in cell viability than single agent, indicating that combination of Rolapitant and targeted drugs can remarkably accelerate synergistic anti-lung cancer activity (Fig. 7D). We next tested the combination therapy of Rolapitant with Gefitinib or Afatinib in A549 xenografts. The growth of tumors in combination treatment with Gefitinib or Afatinib were more significantly suppressed than Gefitinib or Afatinib alone (Fig. 7E). Tumor weight and volume were more dramatically decreased in treatment groups than control mice or Rolapitant alone (Fig. 7F). There was no significant difference in average body weight between the control and treatment groups (Fig. 7G).

In summary, Rolapitant suppresses the growth of human lung cancer cells in vivo and significantly reduces the lung cancer cell viability when combined with targeted drugs, demonstrating that Rolapitant has the potential for development as a new anti-cancer agent.

## Discussion

About 100 DUBs have been identified in humans, which can be divided into six families:: ubiquitin-specific proteases (USPs), ovarian tumor proteases (OTUs), ubiquitin C-terminal hydroxylases (UCHs), Machado-Josephin domain proteases (MJDs), Jab1/Mov34/Mpr1 Pad1 N-terminal+ proteases (JAMMs), motif interacting with ubiquitin-containing novel DUB family proteases (MINDYs), and zinc finger containing ubiquitin peptidase 1 (ZUP1) [42]. DUBs act as oncogenes or tumor suppressors in different types of cancers [43]. OTUD3 plays a context-dependent role in diverse cancer types. OTUD3 could deubiquitylate and stabilize the tumor suppressor PTEN in breast cancer at the protein level. Furthermore, *Otud3* transgenic mice have shown increased PTEN protein levels and reduced susceptibility to breast tumorigenesis [44], while *Otud3* knockout (KO) mice exhibited the opposite trends. However, we recently observed *Otud3* transgenic mice are more susceptible to Kras^G12D^-driven lung cancer, while *Otud3* KO mice are less susceptible [2], indicating an oncogenic role of OTUD3 in lung cancer. OTUD3 is overexpressed in lung cancer, and the increased expression of OTUD3 is associated with short survival and poor prognosis in lung cancer patients [2]. Mechanistically, OTUD3 promotes the proliferation of lung adenocarcinoma through deubiquitylation and stabilization of GRP78 [2]. GRP78, a major ER chaperone, is overexpressed in multiple cancers and involved in promoting tumor growth and metastasis, which can also accelerate protein folding and participate in the unfolded protein response. Consequently, tumor growth and metastasis can be inhibited by targeting cell surface-localized GRP78 in cancers. Therefore, inhibition of OTUD3-GRP78 signaling axis has been proposed as an anti-cancer therapeutic target in lung cancer. In our most recent study, we identified Rolapitant from FDA-approved drugs as an inhibitor of OTUD3. Rolapitant, a high-affinity NK1 receptor antagonist that was approved in September 2015 as a treatment for nausea and vomiting caused by chemotherapy [19], has not been reported in tumors as an antiemetic drug at present. The Rolapitant targets the OTUD3-GRP78 axis and displays DR5-induced anti-lung cancer activity in both in vitro and in vivo models.

Anti-cancer agents can initiate the death receptor–induced (extrinsic) apoptosis, which is one of the most important cytotoxic pathways [45–48]. TRAIL is considered to be an attractive agent for cancer therapy because it can induce apoptosis of cancer cell without causing toxicity [31]. However, most cancer cell lines and primary tumors such as NSCLC develop TRAIL resistance [30], resulting in general treatment failure with advanced cancer patients [49]. Resistance of tumor cells to TRAIL-induced apoptosis continues to be an important factor in the failure of clinical trials, which indicates that only when a TRAIL sensitizer is used can cancer therapy containing TRAIL be effective. There is increasing evidence that TRAIL death receptors (DRs) are dysfunctional in various cancer cell types [50]. Hence, reconstitution of TRAIL receptors in cancer cells is an efficient strategy for the development of biotherapy drugs to conquer TRAIL resistance. Recent studies have shown that TRAIL resistance can be reversed by upregulating DR5 with chemotherapeutic agents [51, 52] and ER stress can trigger the augment of DR5 level in a range of human cancers through the transactivation of the transcription factor CHOP [32]. Moreover, some previous findings demonstrated that CHOP regulated ER stress-induced apoptosis by enhancing the expression of DR5 in some types of human cancer cells. CHOP could trigger the increased expression of DR5, an ER stress inducer, by binding to the 5′ untranslated region of DR5 promoter [32]. This discovery provides clues for developing TRAIL-sensitizing agents. Many ER stress inducers, for example, thapsigargin, have been reported to possess anti-cancer impacts in combination with TRAIL [53]. Nevertheless, the stability and safety of these drugs for the clinical application remain unknown. A promising strategy is to find new chemotherapeutic agents from FDA-approved drugs combined with TRAIL to ensure safety and efficacy in the clinic. In our study, we successfully identified Rolapitant as a potential TRAIL-receptor agonist from FDA-approved drugs library. Our in vitro and in vivo studies provide evidence that Rolapitant regulates ER stress through targeting the OTUD3-GRP78 axis to induce CHOP-DR5 signaling, exerting synergistic lethal effects with TRAIL on lung cancer.

**Fig. 8:**
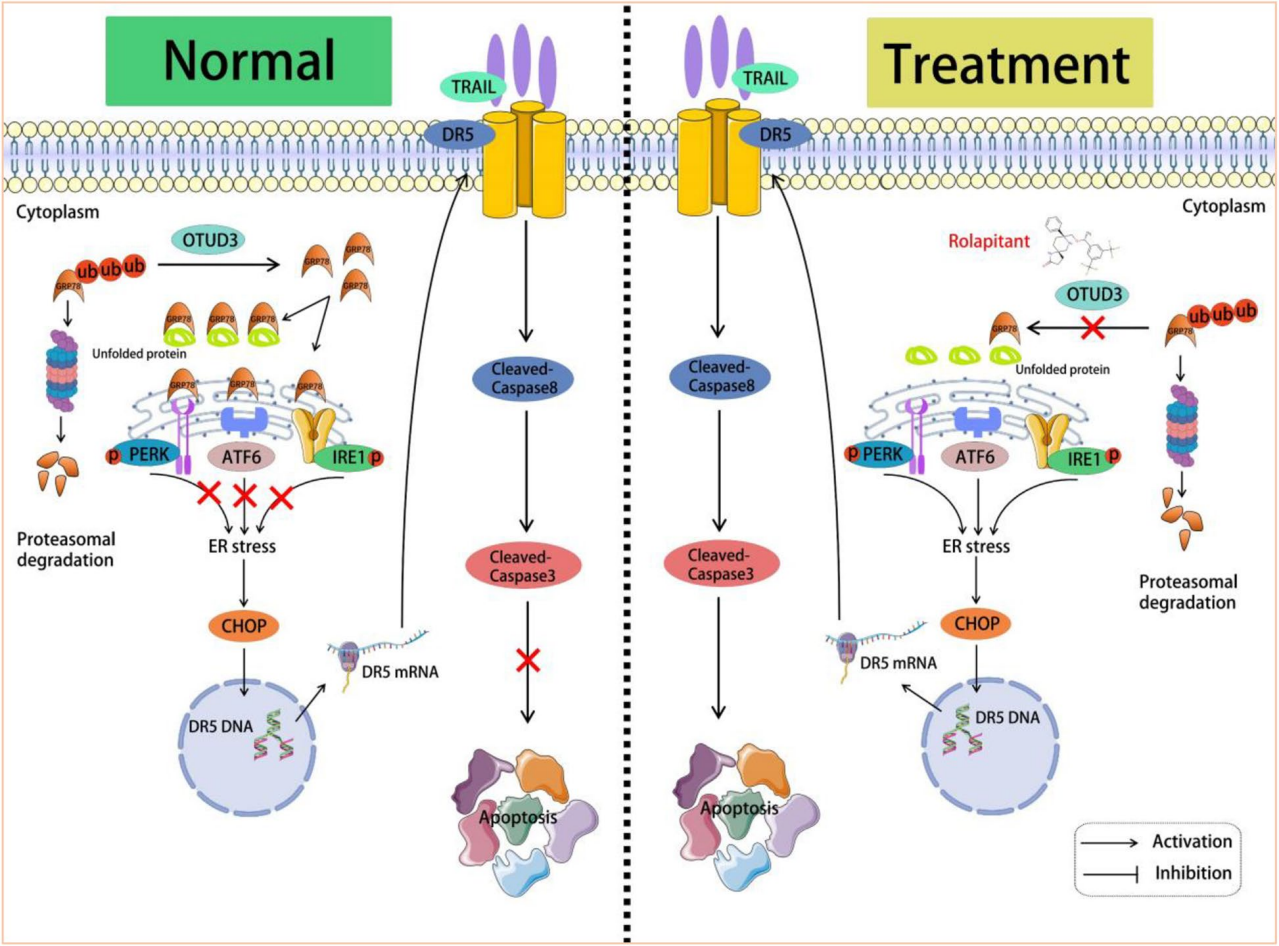
Model of Rolapitant treats lung cancer by targeting deubiquitinase OTUD3

Chaperone proteins are located in the ER and assist with transmembrane transport as well as the correct folding of growth factors and transmembrane receptors [54]. The chaperone binding immunoglobulin protein (BiP) is the major folding protein in the ER, which is known as GRP78 [54]. GRP78 regulates UPR by binding to and inactivating all three ER stress transducers: PERK, IRE1 and ATF6 [55, 56]. GRP78 binds to the misfolded proteins accumulated in the ER and then releases the UPR sensor, resulting in the activation of UPR pathways [57]. On the contrary, UPR can be triggered spontaneously when GRP78 is depleted or inactivated, leading to different physiological consequences [58, 59]. Our previous study showed that OTUD3 stabilized the GRP78 protein in lung cancer cells [2]. But we found that when Rolapitant was added to lung cancer cells, Rolapitant inhibits the deubiquitinating activity of OTUD3, thereby promoting GRP78 ubiquitination and resulting in its degradation. Furthermore, Rolapitant leads to GRP78 separation from the three ER stress transducers PERK, IRE1, and ATF6, activating the three UPR sensors and triggering ER stress. The resulting ER stress upregulates CHOP signaling and promotes the transcriptional activity of DR5 (Fig. 8).

These findings imply that Rolapitant promotes DR5-induced apoptosis through causing ER stress. Although ER stress can promote the transcriptional activity of GRP78, OTUD3 loses the ability to stabilize GRP78 in the presence of Rolapitant. For the first time, we demonstrated that the inhibition of OTUD3-GRP78 axis with Rolapitant induces ER stress-DR5-mediated extrinsic apoptosis to significantly inhibit the growth of human lung cancer.

## Conclusion

Based on our data, we propose that Rolapitant directly inhibit the deubiquitinating activity of OTUD3 through binding to OTUD3 to cause the downregulation of GRP78 protein levels. The dissociation of GRP78 from ER stress sensors leads to the activation of the PERK, IRE1, and ATF6 signaling pathway, triggering ER stress. Then, the ER stress promotes DR5 expression in a CHOP-dependent manner. This finding underlines the potential of Rolapitant targeting the OTUD3-GRP78 axis in the clinical treatment of TRAIL-resistant lung cancers.

### Supplementary Information

**Additional file 1.**

## Data Availability

The data that support the fndings of this study are available from the corresponding author upon reasonable request.
