# Supplementary material for: Rolapitant treats lung cancer by targeting deubiquitinase OTUD3
Source: Cell Commun Signal. 2024 Mar 27;22:195. doi: 10.1186/s12964-024-01519-8 (PMC10967203; doi:10.1186/s12964-024-01519-8)
Supplement: Supplementary file 1 — Additional file 1. [file 12964_2024_1519_MOESM1_ESM.pdf]

Figure3

Fig.3A

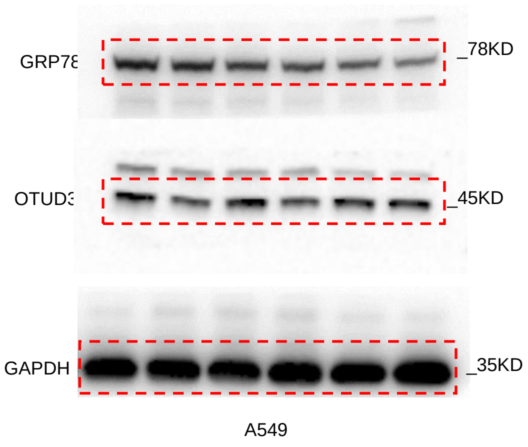

Fig.3B

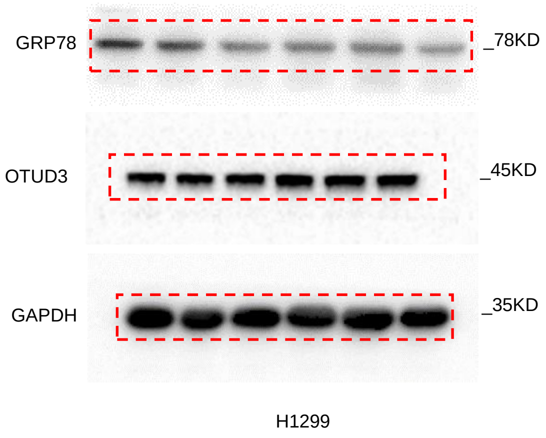

Fig.3C

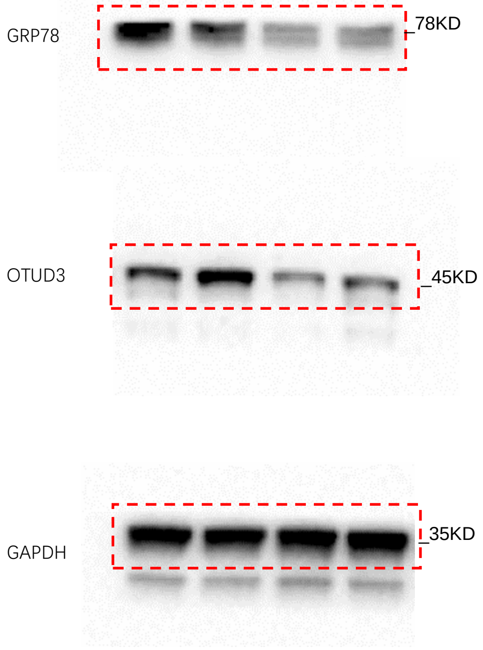

Fig.3D

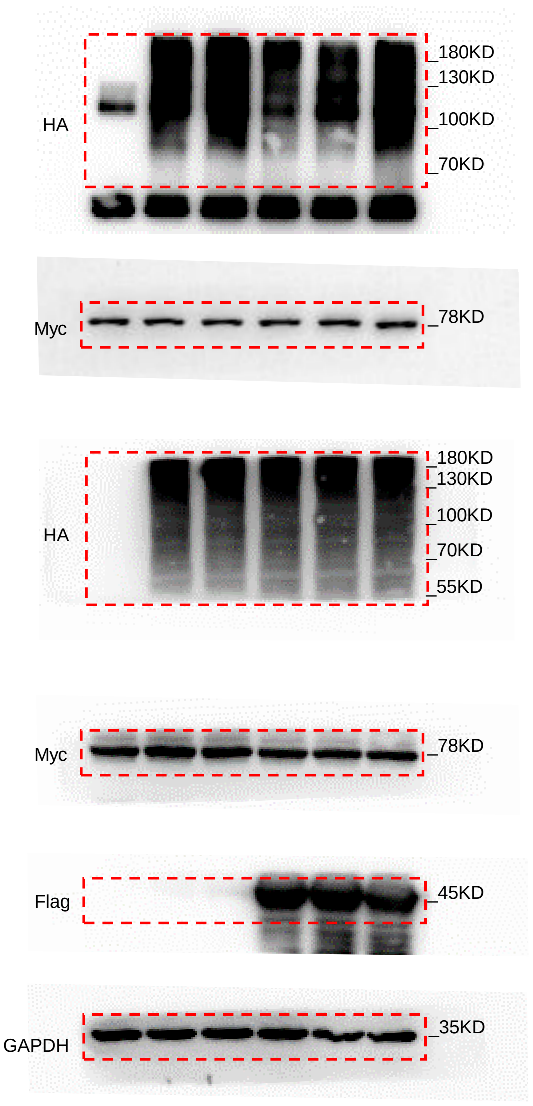

Figure3

Fig.3E

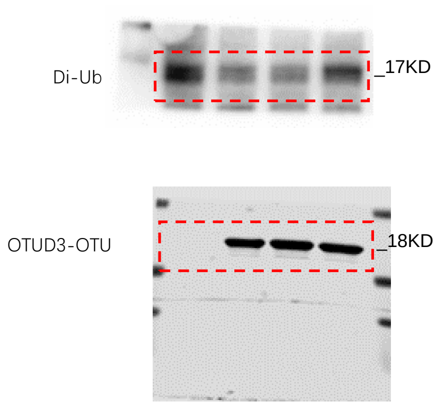

Fig.3F

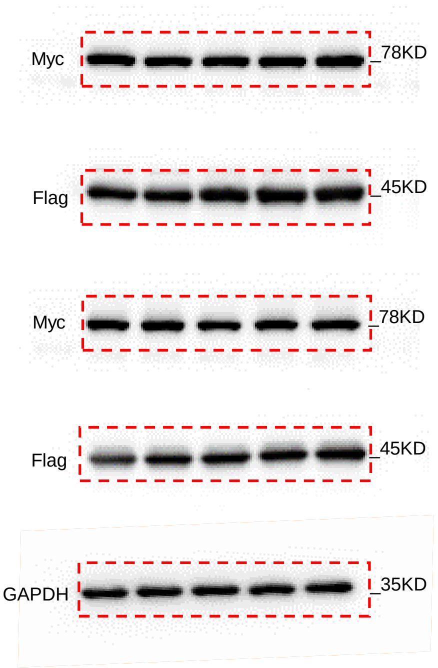

Fig.3G

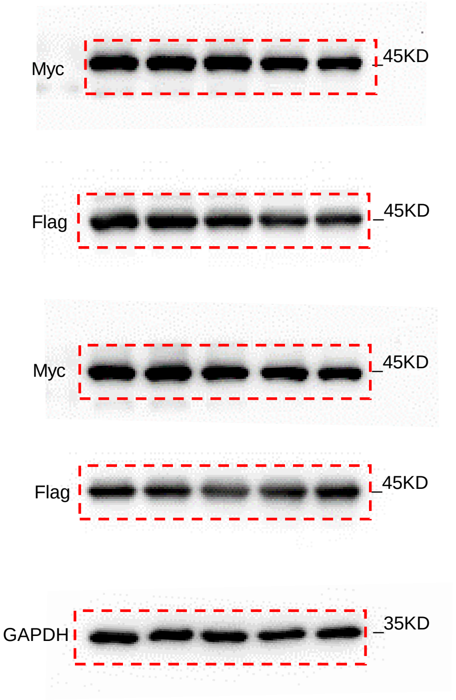

Fig.3H

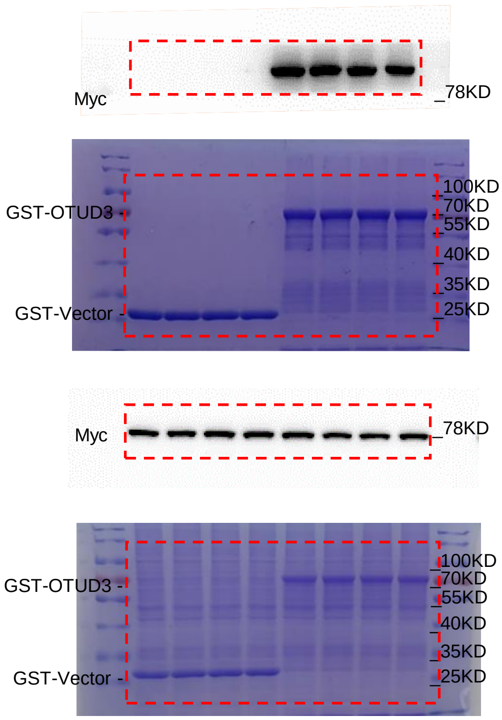

Fig.3I

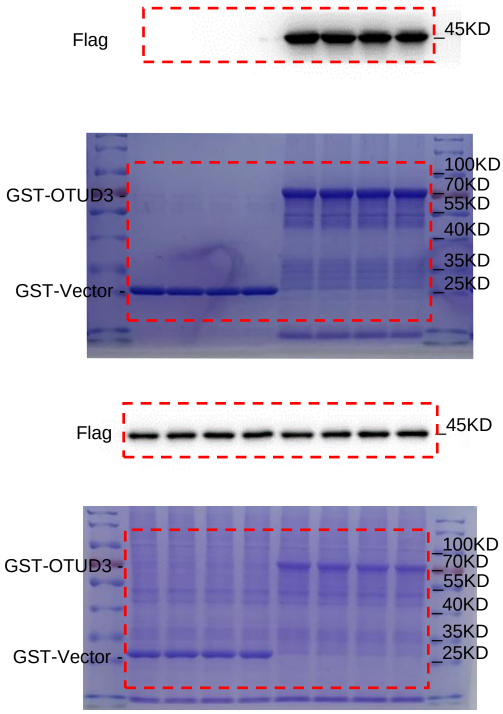

Figure 4

Fig4 E

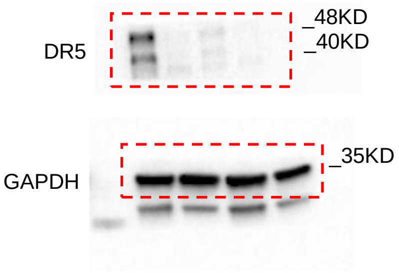

A549

Fig4 E

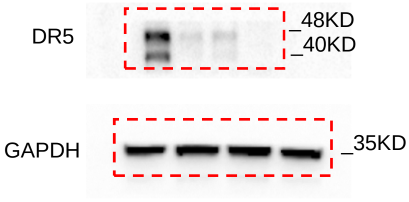

H1299

Fig4 H

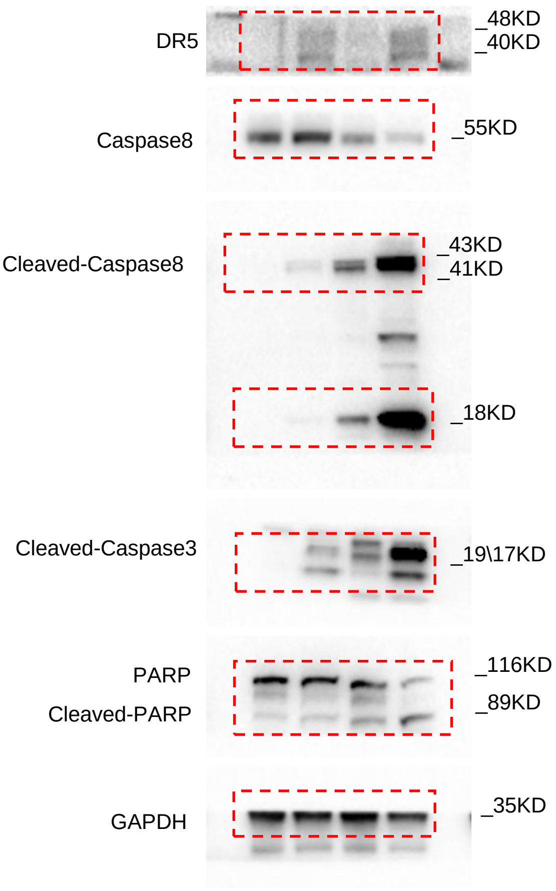

H1299

Fig4 H

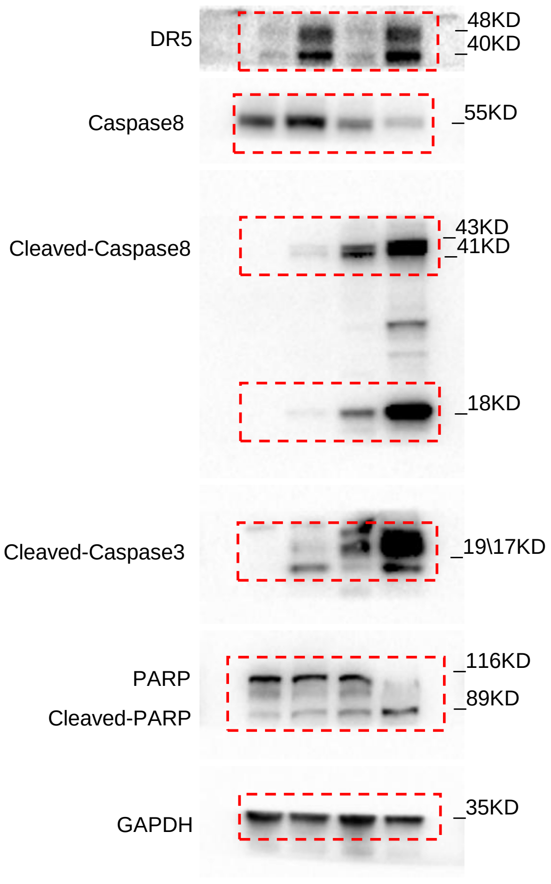

A549

Figure 5

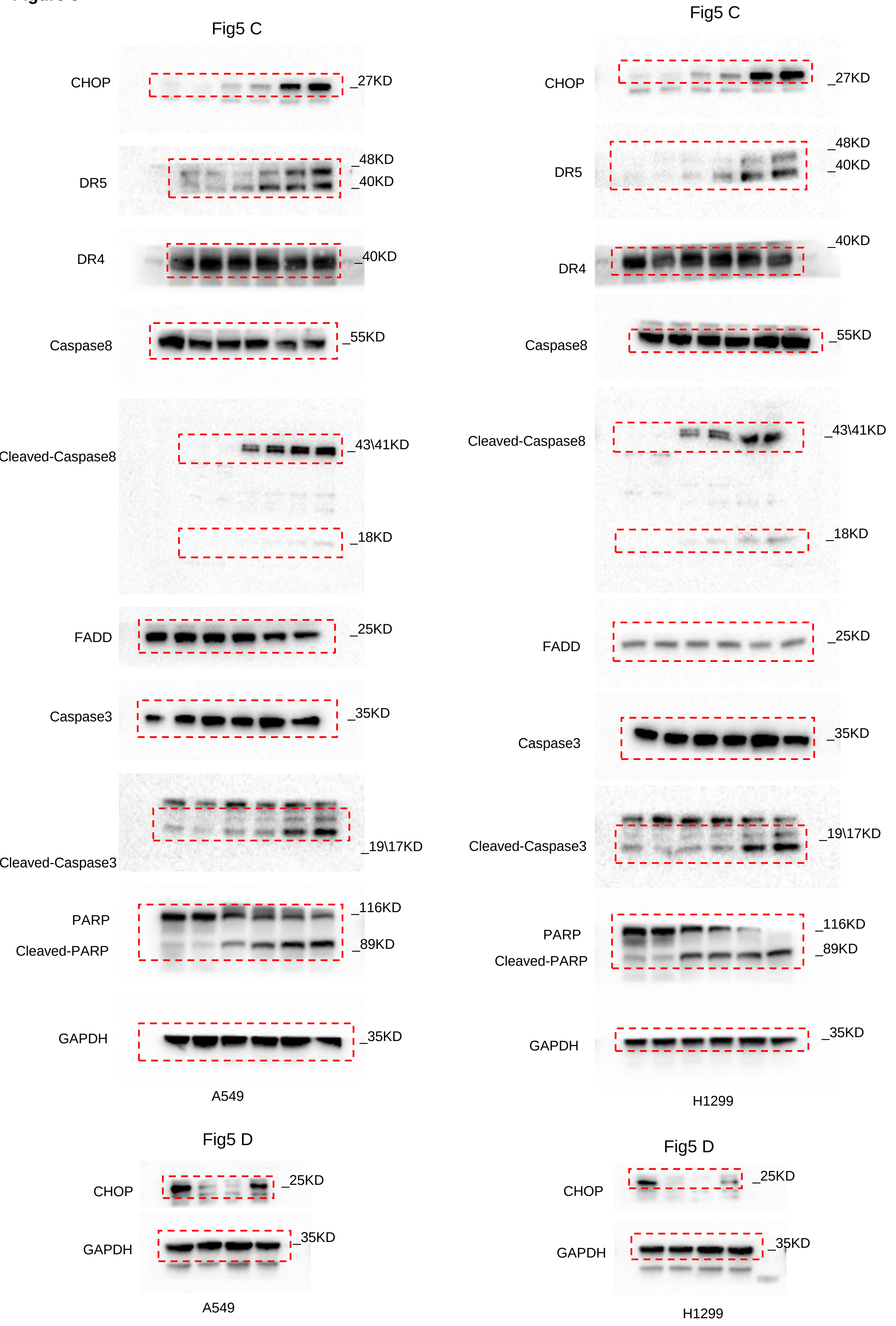

Fig5 C
CHOP
_27KD
DR5
_48KD
_40KD
DR4
_40KD
Caspase8
_55KD
Cleaved-Caspase8
_43\41KD
_18KD
FADD
_25KD
Caspase3
_35KD
Cleaved-Caspase3
_19\17KD
PARP
Cleaved-PARP
_116KD
_89KD
GAPDH
_35KD
A549
Fig5 C
CHOP
_27KD
DR5
_48KD
_40KD
DR4
_40KD
Caspase8
_55KD
Cleaved-Caspase8
_43\41KD
_18KD
FADD
_25KD
Caspase3
_35KD
Cleaved-Caspase3
_19\17KD
PARP
Cleaved-PARP
_116KD
_89KD
GAPDH
_35KD
H1299
Fig5 D
CHOP
_25KD
GAPDH
_35KD
A549
Fig5 D
CHOP
_25KD
GAPDH
_35KD
H1299

Fig5 E

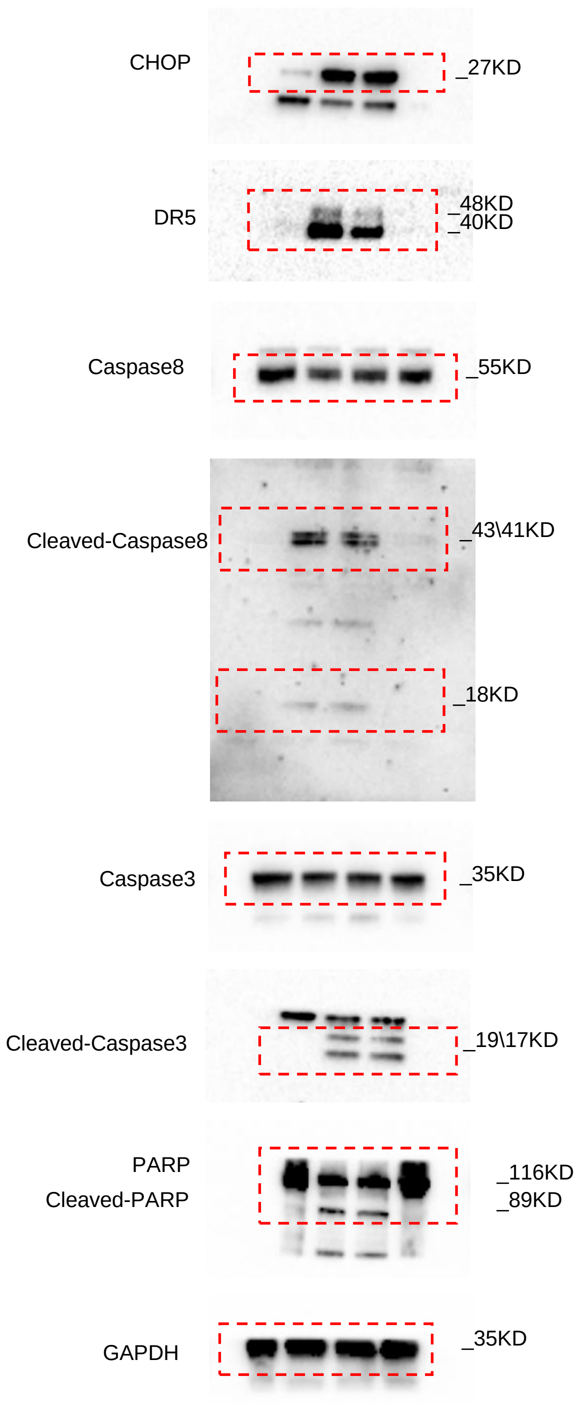

A549

Fig5 E

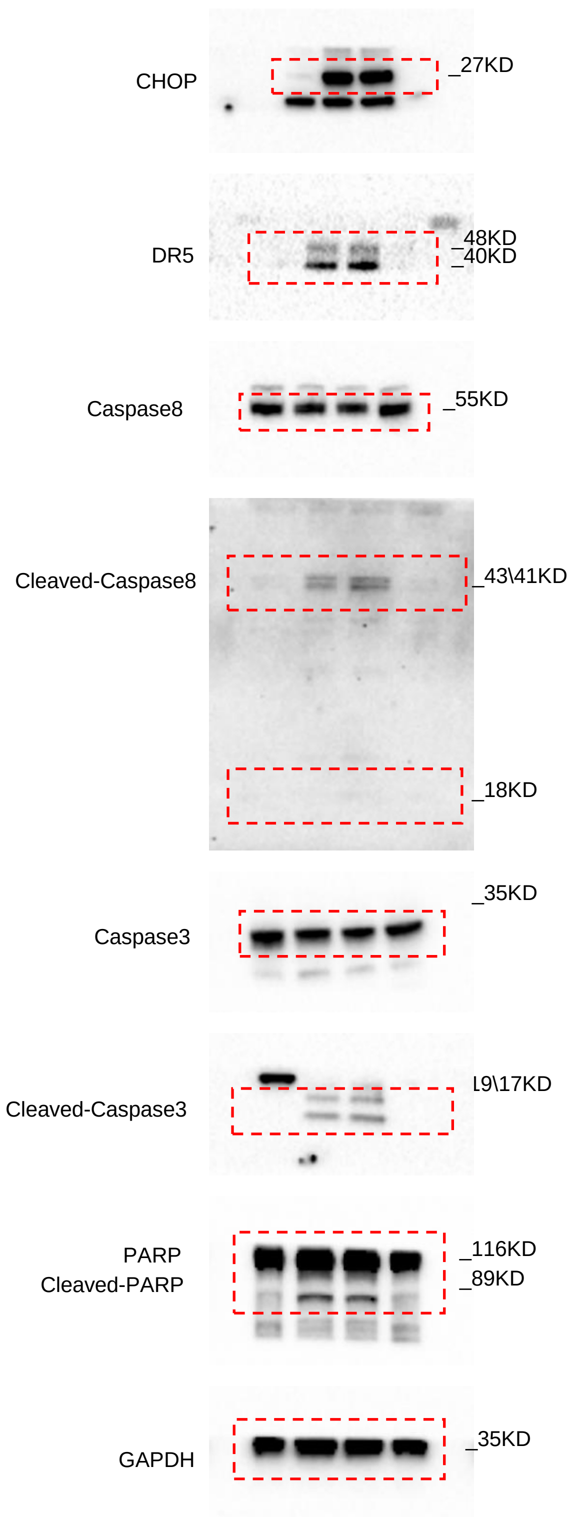

H1299

Fig5 F

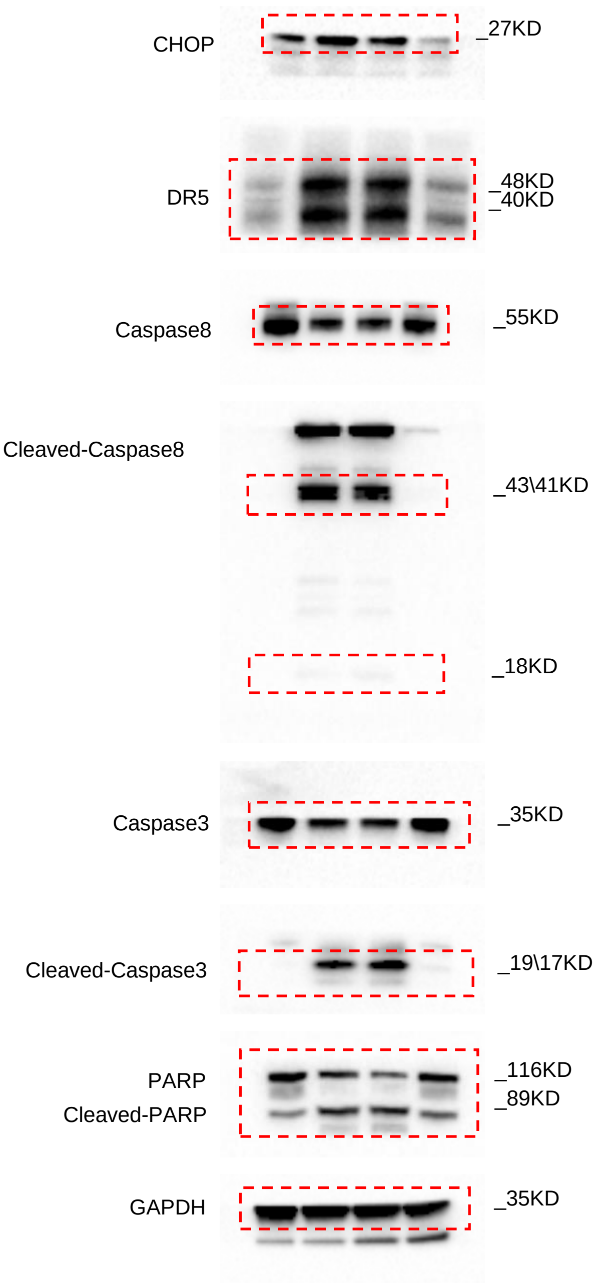

A549

Fig5 F

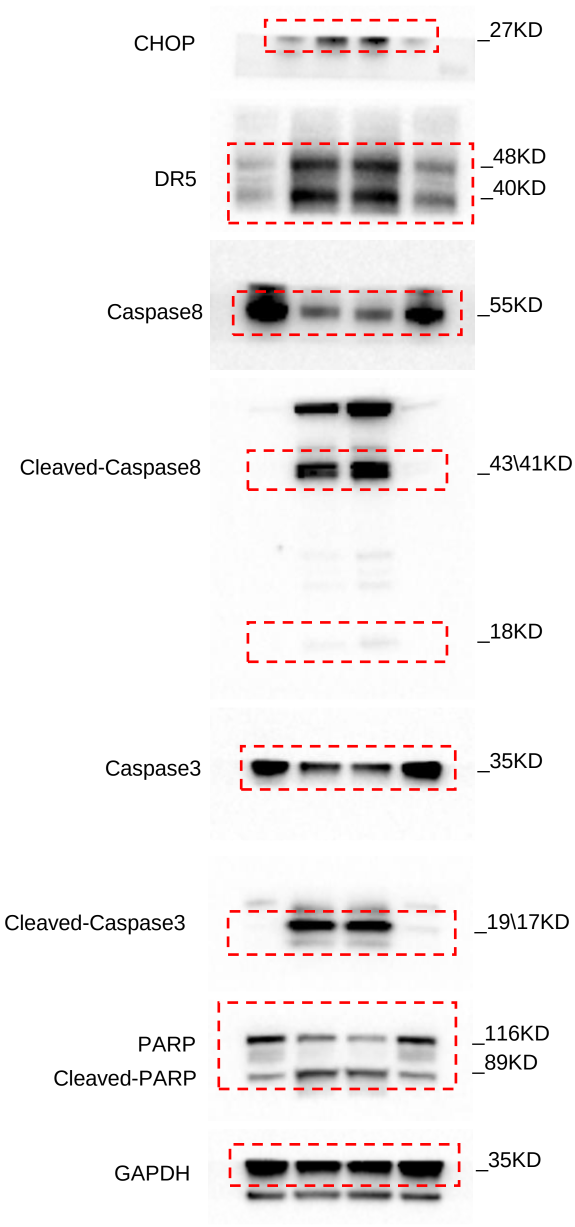

H1299

Fig6 A

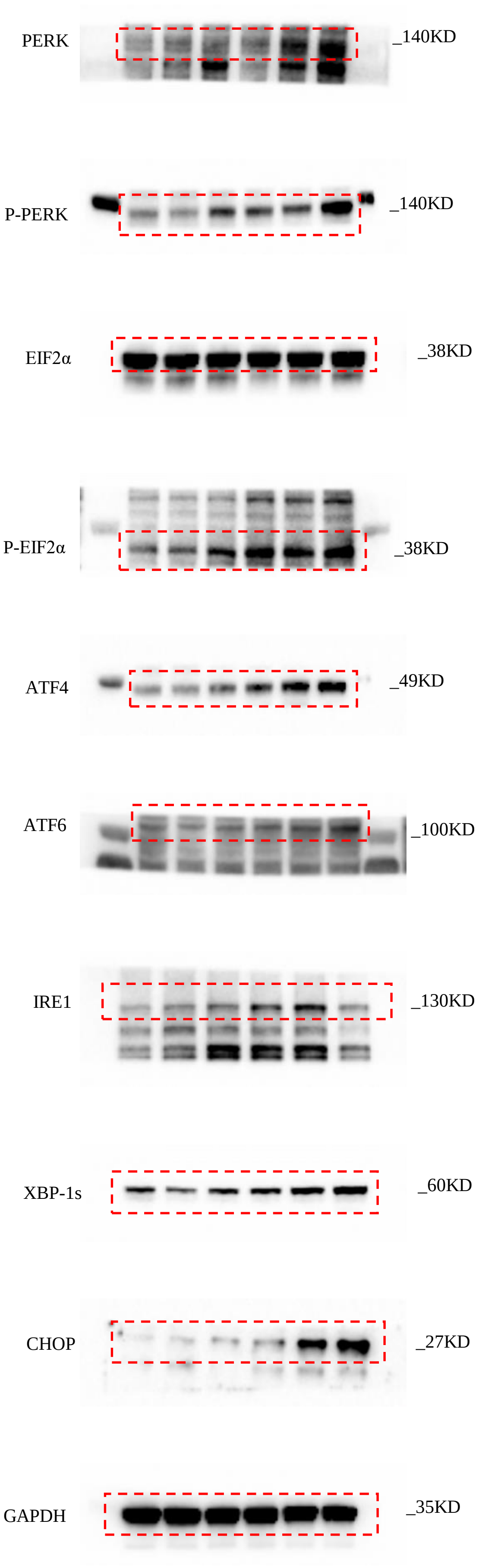

A549

Fig6 A

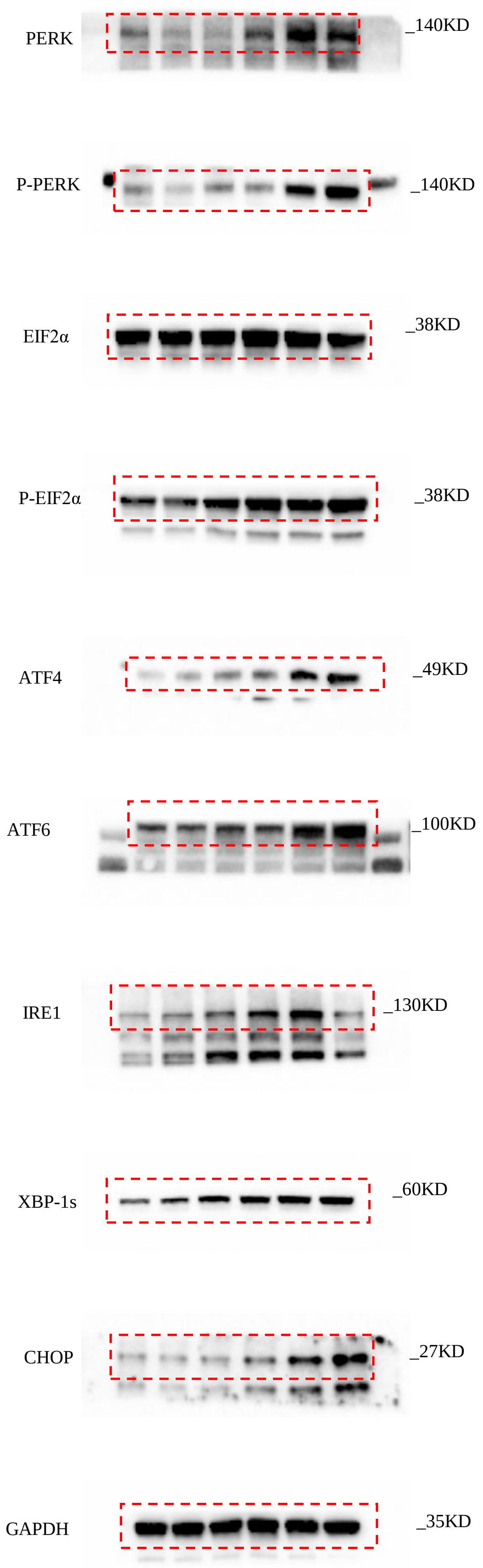

H1299

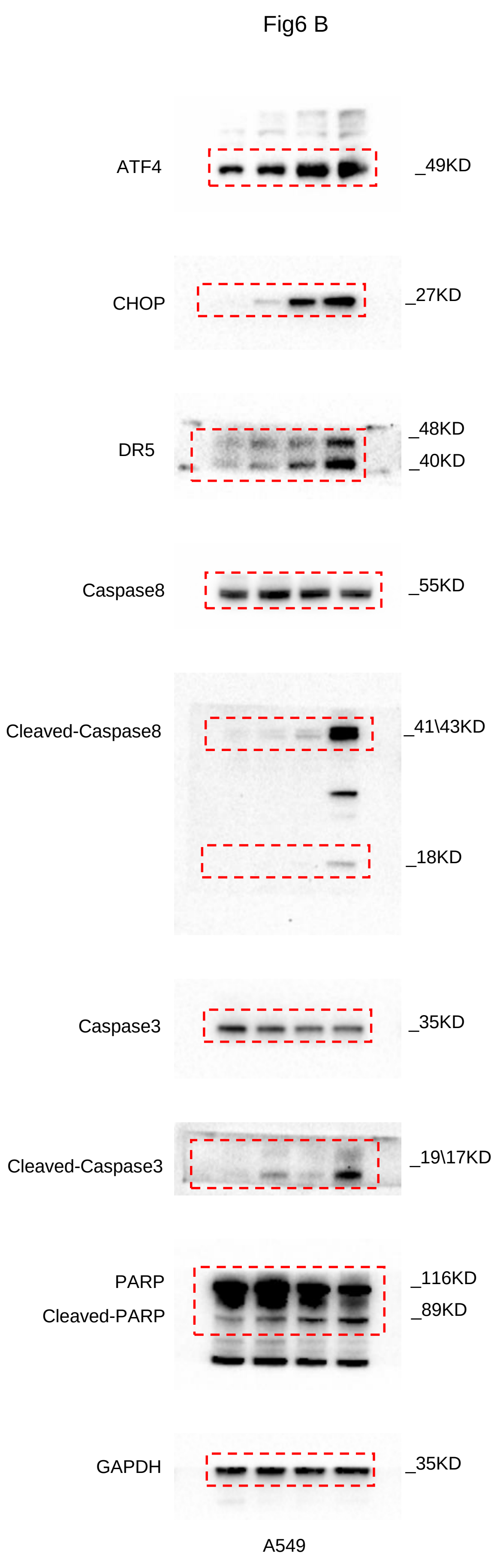
Fig6 B
ATF4
_49KD
CHOP
_27KD
DR5
_48KD
_40KD
Caspase8
_55KD
Cleaved-Caspase8
_41\43KD
_18KD
Caspase3
_35KD
Cleaved-Caspase3
_19\17KD
PARP
Cleaved-PARP
_116KD
_89KD
GAPDH
_35KD
A549

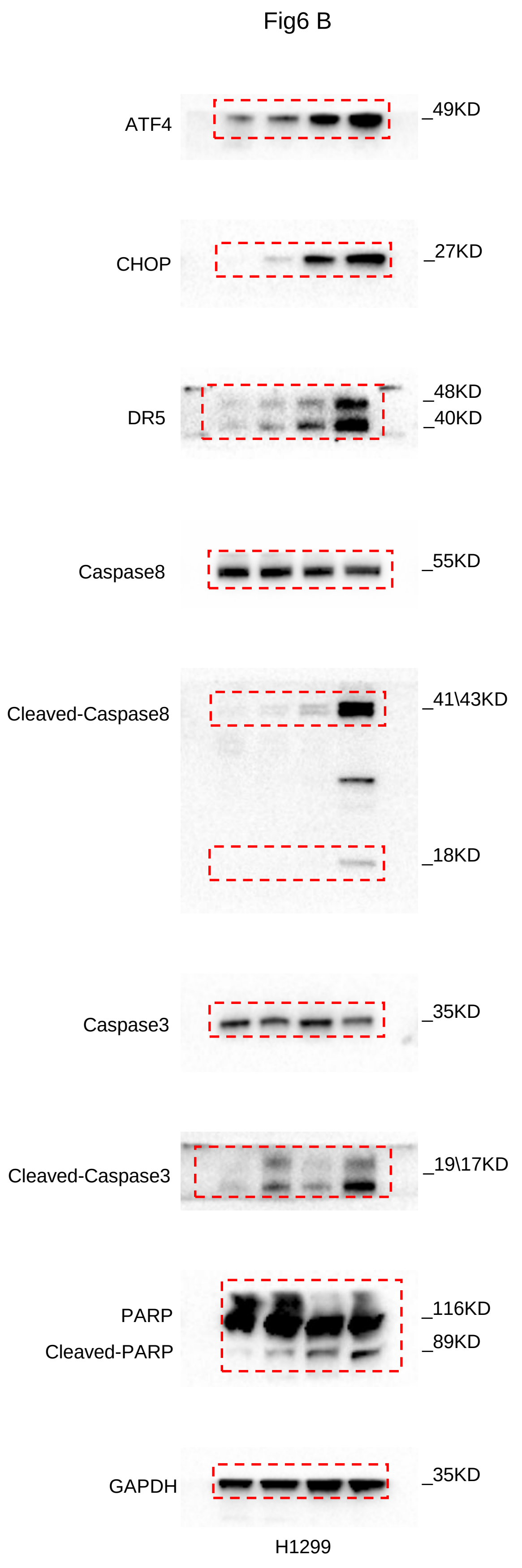
Fig6 B
ATF4
_49KD
CHOP
_27KD
DR5
_48KD
_40KD
Caspase8
_55KD
Cleaved-Caspase8
_41\43KD
_18KD
Caspase3
_35KD
Cleaved-Caspase3
_19\17KD
PARP
Cleaved-PARP
_116KD
_89KD
GAPDH
_35KD
H1299

Fig6 C

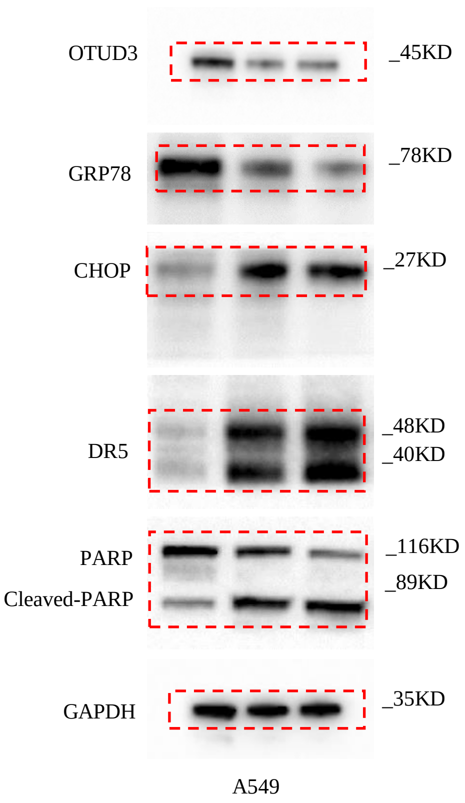
OTUD3
_45KD
GRP78
_78KD
CHOP
_27KD
DR5
_48KD
_40KD
PARP
Cleaved-PARP
_116KD
_89KD
GAPDH
_35KD
A549

Fig6 C

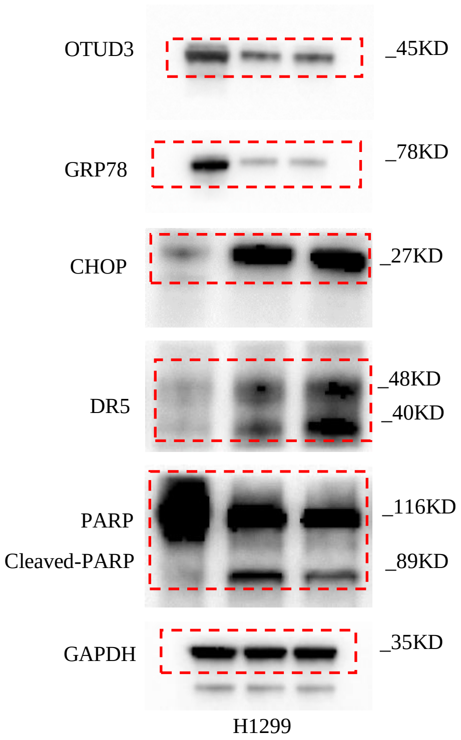
OTUD3
_45KD
GRP78
_78KD
CHOP
_27KD
DR5
_48KD
_40KD
PARP
Cleaved-PARP
_116KD
_89KD
GAPDH
_35KD
H1299

Fig6 D

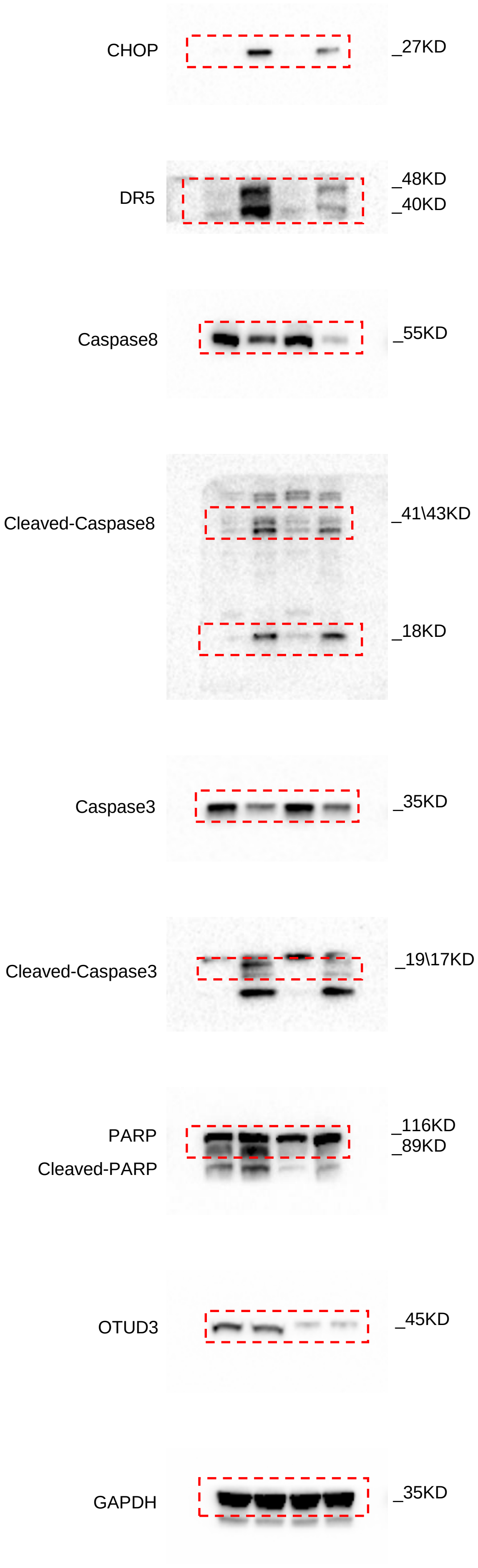

A549

Fig6 D

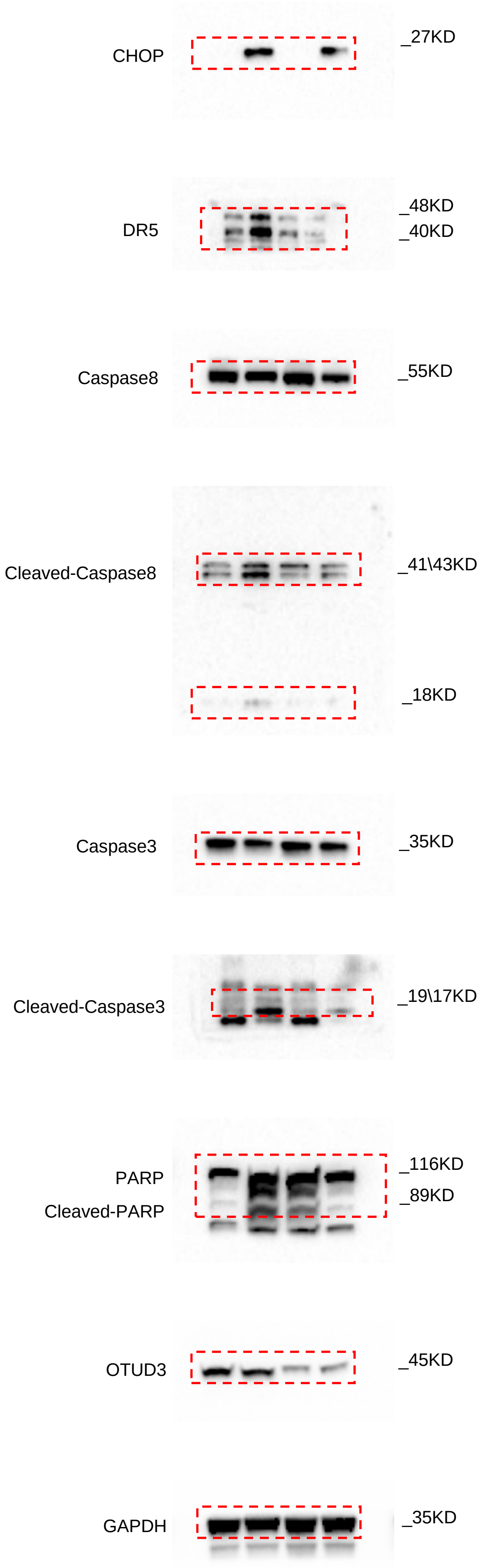

H1299

Fig6 E

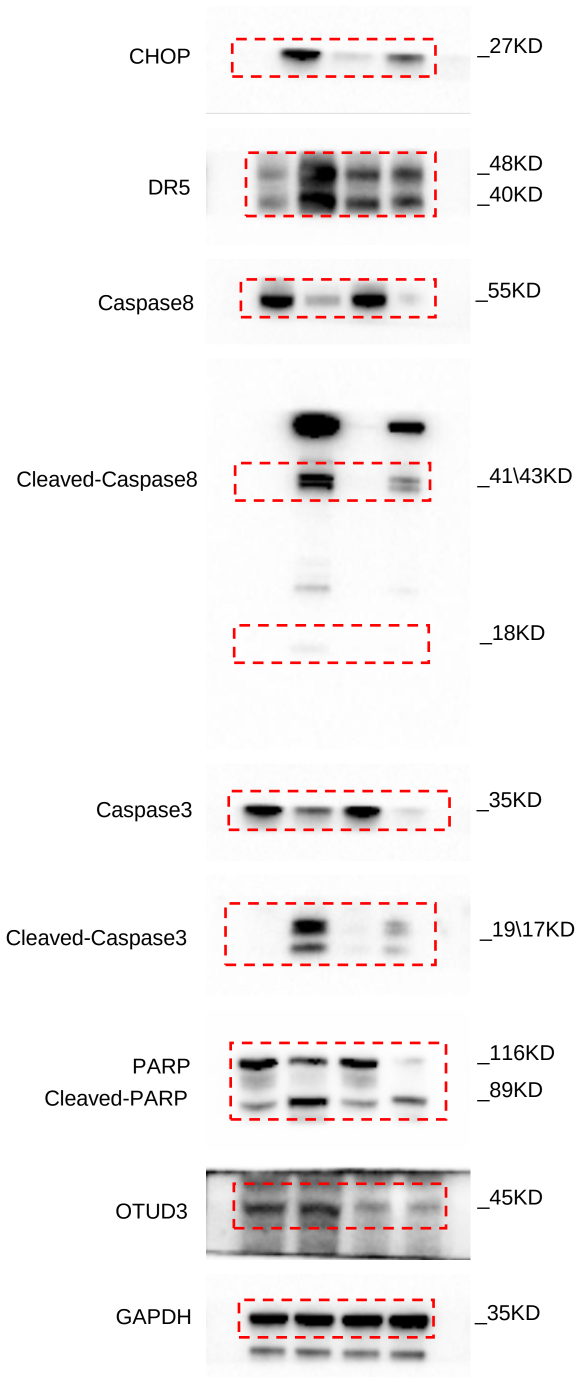

A549

Fig6 E

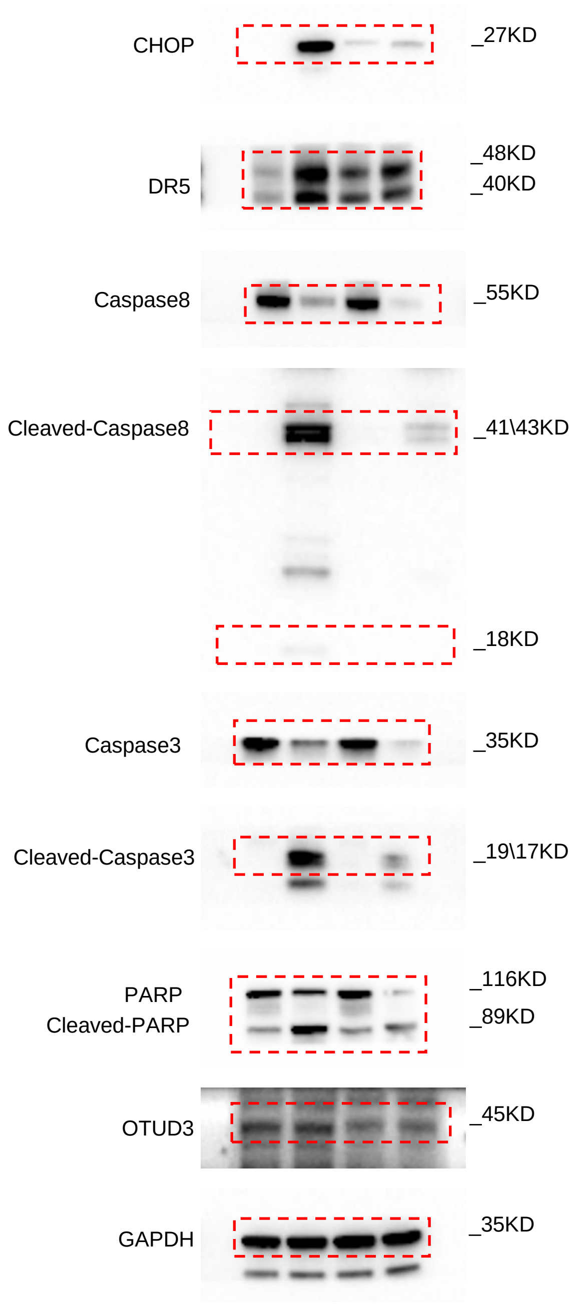

H1299
